# Analyzing alternative splicing in Alzheimer’s disease postmortem brain: a cell-level perspective

**DOI:** 10.3389/fnmol.2023.1237874

**Published:** 2023-09-20

**Authors:** Mohammad-Erfan Farhadieh, Kamran Ghaedi

**Affiliations:** Department of Cell and Molecular Biology and Microbiology, Faculty of Biological Sciences and Technology, University of Isfahan, Isfahan, Iran

**Keywords:** Alzheimer’s disease, alternative splicing, alternative polyadenylation, differential transcript usage, single cell RNA sequencing

## Abstract

Alzheimer’s disease (AD) is a neurodegenerative disease with no effective cure that attacks the brain’s cells resulting in memory loss and changes in behavior and language skills. Alternative splicing is a highly regulated process influenced by specific cell types and has been implicated in age-related disorders such as neurodegenerative diseases. A comprehensive detection of alternative splicing events (ASEs) at the cellular level in postmortem brain tissue can provide valuable insights into AD pathology. Here, we provided cell-level ASEs in postmortem brain tissue by employing bioinformatics pipelines on a bulk RNA sequencing study sorted by cell types and two single-cell RNA sequencing studies from the prefrontal cortex. This comprehensive analysis revealed previously overlooked splicing and expression changes in AD patient brains. Among the observed alterations were changed in the splicing and expression of transcripts associated with chaperones, including *CLU* in astrocytes and excitatory neurons, *PTGDS* in astrocytes and endothelial cells, and *HSP90AA1* in microglia and tauopathy-afflicted neurons, which were associated with differential expression of the splicing factor *DDX5*. In addition, novel, unknown transcripts were altered, and structural changes were observed in lncRNAs such as *MEG3* in neurons. This work provides a novel strategy to identify the notable ASEs at the cell level in neurodegeneration, which revealed cell type-specific splicing changes in AD. This finding may contribute to interpreting associations between splicing and neurodegenerative disease outcomes.

## 1. Introduction

Alzheimer’s disease (AD), the most common cause of dementia, is a progressive degenerative disorder that attacks the brain’s cells resulting in loss of memory, thinking, and language skills, and changes in behavior with extracellular β-amyloid (Aβ) aggregation and neurofibrillary tangles (NFT) formed by hyperphosphorylated tau (Knopman et al., 2021). The global number of individuals with AD is estimated to be 32 million. At the same time, prodromal and preclinical AD comprised 416 million persons worldwide, or 22% of all people aged 50 and over (Gustavsson et al., 2023). AD is a complex and multifaceted disease, and much is still unknown about its causes and progression (Herrup, 2021). As a result, there are many hypotheses about the disease, but no single answer or successful cure (Alves et al., 2021).

A single gene can create numerous proteins through alternative splicing (Gabut et al., 2011). It is an essential mechanism of gene regulation in the brain and is known to be altered in aging and neurodegeneration (Chabot and Shkreta, 2016; Deschenes and Chabot, 2017). Alternative splicing is mediated by a ribonucleoprotein complex called the spliceosome, which consists of five small nuclear ribonucleoprotein subunits and several protein cofactors (Matera and Wang, 2014). Because of the short and degenerate splicing sites in higher eukaryotes, the spliceosome usually requires RNA binding proteins (RBP) called splicing factors (SF) to identify exons accurately (Biamonti et al., 2021). Generally, seven basic types of alternative splicing events (ASEs) have been identified, including alternative 3′-splice site (A3SS), alternative 5′-splice site (A5SS), mutually exclusive exons (MXE), intron retention (RI), exon skipping (SE), alternative polyadenylation (APA), and alternative promoter (Bhadra et al., 2020). Recent transcriptomic studies have found several ASEs to be disrupted in AD, such as *MBP*, *ABCA7*, *APP*, *CLU*, *PICALM*, and *PTK2B* (Raj et al., 2018; Yang et al., 2021). Also, it is observed that the U1 snRNP deposition with NFT in postmortem brains of AD patients (Bai et al., 2013; Hales et al., 2014), MAPT transgenic mice (Vanderweyde et al., 2016; Maziuk et al., 2018), and in laboratory conditions (Bishof et al., 2018). As alternative splicing is a cell type-specific process (Zhang et al., 2016), and the balance of cell types is disturbed in AD so that there are fewer neurons and more glia (Penney et al., 2020), studying ASEs at the cellular level becomes necessary. Sharing of RNA sequencing (scRNA-seq) data of AD patients and control with single cell RNA sequencing data of Allen brain atlas of Brodmann area 22 suggested that there is cell-type differential transcript usage (DTU) pattern for APP and BIN1 (Marques-Coelho et al., 2021). ScRNA-seq data analyses a showed that Sipa1l1 transcripts were changed differently between the brains of APOE-deficient transgenic mice and control mice (Weller et al., 2022).

**Figure 1:**
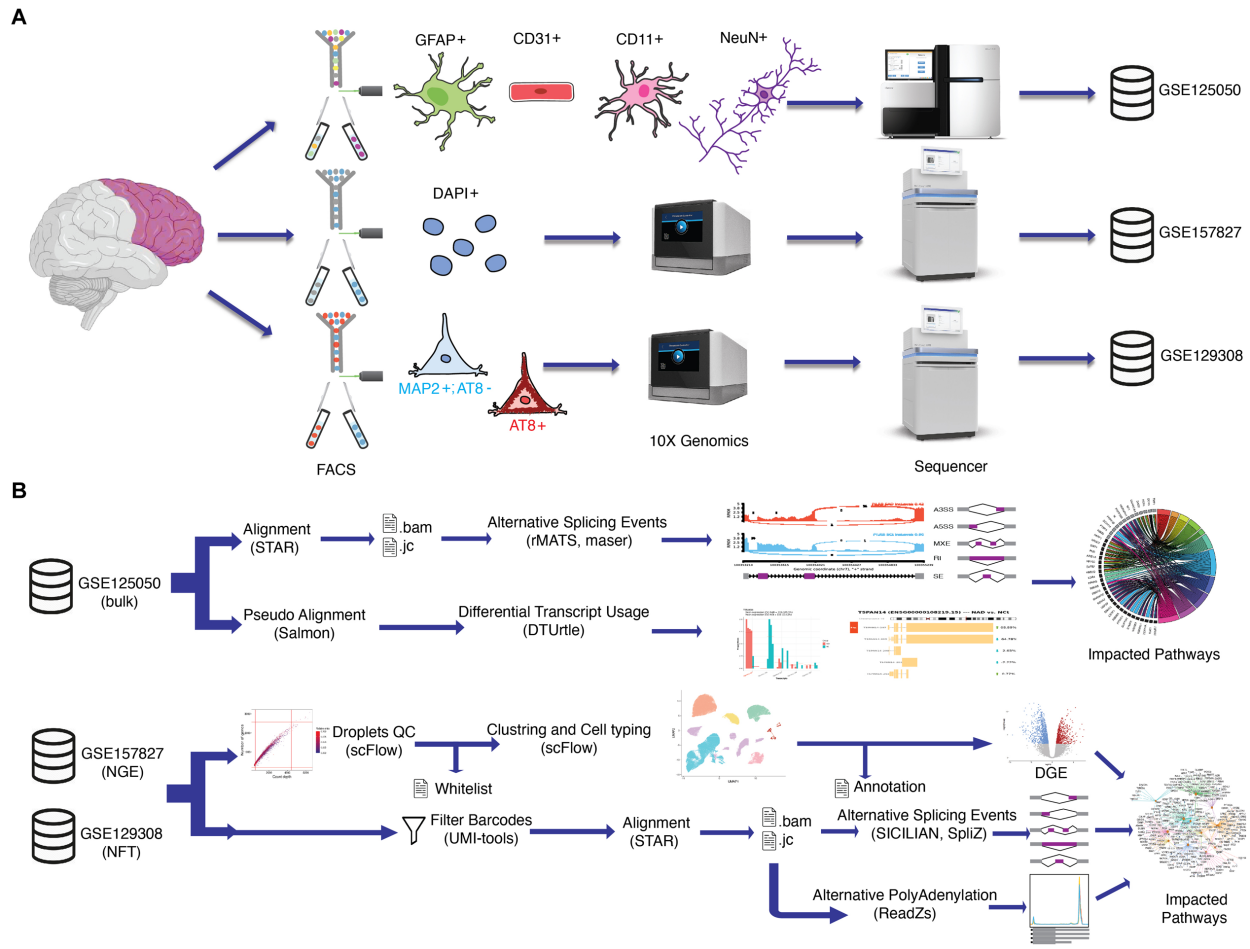
Schematic workflow of the methodology. **(A)** Dataset preparation in previous studies. In GSE125050 (bulk), the prefrontal cortex (PFC) region of postmortem brains was sorted by FACS into four cell groups, including astrocyte (GFAP+), endothelial (CD31+), microglia (CD11+) and neuron (NeuN+). Then groups were sequenced with bulk RNAseq protocol. In GSE157827 (NGE), single nuclei of PFC were isolated by gradient centrifugation, FACS, and 10X Genomix, respectively. In GSE129308 (NFT), single PFC somas were mechanically dissociated by Potter-Elvehjem grinder without enzymes or detergents and gradient centrifugation. Somas were sorted to neurofibrillary tangles (NFT)_bearing neurons (AT8+) and NFT_free neurons (MAP2+/AT8−) by FACS and followed scRNAseq protocol. **(B)** Datasets obtained from GEO and SRA. GSE125050 data is used for alternative splicing events analysis and differential transcript usage analyses, and significant alterations are used for impacted pathway analyses. Both GSE157827 and GSE129308 are analyzed in the same workflow separately. GSE157827 is used for alternative splicing events and alternative polyadenylation analyzes all CNS cell types between two Alzheimer’s disease stages and control groups, while GSE129308 is used for ASE and APA analyzes between NFT_bearing neurons and NFT_free neurons in same individuals. Robust statistics tools, including STAR, SICILIAN, SpliZ, and ReadZs, were used to detect differential exon junctions and 3′ end peaks. A new protocol was designed to reduce biological noises in final results, which includes doublet, damaged cells, and empty droplets by determining true cell barcodes in a whitelist file. UMI_tools used the whitelist file for the filtration of fastq files. Finally, impacted pathways were analyzed.

In this study, we analyzed transcripts variations at the cell level in the postmortem prefrontal cortex of AD patients and control patients without neuropathological or neuropsychiatric disorders (Supplementary Table S1) to identify notable ASEs in AD. To do this, we took advantage of three available transcriptomics datasets (Figure 1A). GSE125050 results from bulk RNA sequencing of sorted brain cells in four groups, including astrocytes, endothelial cells, microglia, and neurons (Srinivasan et al., 2020). GSE157827 dataset consists of single nucleus RNA sequencing of neurons, glial cells, and endothelial cells (NGE). Individuals in the NGE dataset were grouped based on the Braak stage in the control group with 0, Alzheimer’s disease with mild severity (ADM) with 3 or 4, and Alzheimer’s disease with high severity (ADH) with 5 or 6 scores (Lau et al., 2020). GSE129308 dataset includes transcriptomic data of single soma RNA sequencing which is grouped based on NFT’s presence in somas (Otero-Garcia et al., 2022). Single-cell data allow the identification of ASEs in rare cell types or subpopulations that may be overlooked in bulk analysis, thereby contributing to a more comprehensive understanding of splicing patterns (Joglekar et al., 2023). Despite the challenges posed by the short reads and 3′ bias in the 10x Genomics’ scRNA-seq data (Westoby et al., 2020), a good understanding of splicing changes can still be achieved due to the presence of a large number of junctional reads in the datasets and the application of a robust statistical approach (Figure 1B). Furthermore, due to the strong correlation between splicing changes and Braak NFT stages in AD (Arizaca Maquera et al., 2023), the utilization of NFT and NGE single-cell data can provide a broader view of ASEs in PFC neuronal subtypes based on NFT pathogenesis and ASEs in different brain cell types based on Braak stages, respectively. A broad identification of ASEs can be gained by analyzing splicing alterations in various cell populations at different stages of AD progression. We also analyzed differential gene expression (DGE) and revealed ontology cell type-specific.

## 2. Materials and methods

### 2.1. Datasets and reference genome

The raw fastq files of GSE125050 (bulk study), GSE157827 (NGE study), and GSE129308 (NFT study) were demultiplexed from the downloaded sra files on short read archive (SRA) database. The metadata of samples was obtained from the gene expression omnibus (GEO) database. The GENCODE version 39 of GRCh38 annotations and sequences files include gff3,^1^ gtf,^2^ bed,^3^ DNA fasta,^4^ cDNA,^5^ and protein domain annotation^6^ files were used for alignment and annotation.

### 2.2. Bulk RNA-seq reads QC and alignment

The raw fastq files were qualified by FastQC (v 0.11.9) and multiQC (v 1.13.0), then trimmed by Trim Galore (v 0.6.7) with 20 as a minimum Phred quality score. The trimmed fastq files were aligned to the reference genome by STAR (v 2.7.10a) with defaults (Dobin et al., 2013). The quality of aligned bam files was controlled with Qualimap (v 2.2.2) and multiQC. The trimmed fastq files also were used to generate transcript expression matrices with the transcriptomic index of reference sequences by Salmon (v 1.7.0; Patro et al., 2017).

### 2.3. ASE analyzes of bulk RNA-seq data

Alternative splicing analyses were performed on the aligned bam files to measure PSI with rMATS (v 4.1.2) and were plotted with rmats2sashimiplot (Shen et al., 2014). Significant ASEs in five categories, including A3SS, A5SS, MXE, RI, and SE, were identified with average coverage >5, delta PSI >10%, and *p* adjusted value (*p.adj*) <0.05 by maser (v 1.12.1) and rtracklayer (v 1.54.0) in R (v 4.2.1; Lawrence et al., 2009; Lu et al., 2022).

### 2.4. DGE and DTU analyzes of bulk RNA-seq data

DGE analysis was performed by DESeq2 on the transcriptome quantification matrix in R (Love et al., 2014). Using import_counts function, which uses the teximport R library in the background. The cutoff *p.adj* was 0.05, and the cutoff fold change was 2. DTU analysis was performed using the R package DTUrtle (Tekath and Dugas, 2021). First, the transcript to gene map was created with the one_to_one_mapping function using the gtf file to annotate each transcript to a single gene and transcript id. Then the statistical analysis was done with DRIMseq, a DTU-specialized statistical framework using the Dirichlet multinomial model inside the DTUrtle package, by defining the groups. The minimum total gene expression must be 5 for at least 50% of the samples of the smallest group. After the dturtle object was generated, to obtain important genes, a post-hoc filter with a value of 0.1, which means more than 10 % changes between transcripts of gene expression, and an overall false discovery rate (OFDR) threshold of 0.05 was used. An overview of significant transcripts was visualized through the Gviz package and the annotated gtf file of the reference genome in R.

### 2.5. Quality control of scRNA-seq droplets

The feature barcode matrices were downloaded from GEO and read by Seurat (v 4.1.0) and SingleCellExperiment (v 1.16.0) in R (Amezquita et al., 2020; Hao et al., 2021). Quality control was performed separately on each sample with functions of the scFlow library (v 0.7.1) in R (Khozoie et al., 2021). The droplets with less than 500 RNA molecules were excluded. Also, the droplets with less than 200 features and genes were excluded, while for a higher feature filter criterion, an adaptive threshold was estimated in each sample that was four times the deviation of the median absolute value above the median feature number in each sample. The droplets with more than 10 % of the whole transcriptome were mitochondrial genes also removed. Only genes with at least one count in three drops per sample were retained. Finally, the identification of multiple droplets was performed using DoubletFinder in the scFlow package using ten principal components (PC) based on 2000 variable features and a pK value of 0.005. Then, the saved barcodes of each sample were recorded in a text file called white list.

### 2.6. scRNA-seq clustering

Each study sample’s modified feature barcode matrices were merged into a SingleCellExperiment object using LIGER (v 1.1.0; Welch et al., 2019). The k value was optimized at 30, and the lambda value at five. Three thousand genes were used in the integration process. The convergence threshold was 0.0001, and the maximum number of block coordinate descent iterations was 100. The Leyden method did the clustering using the resolution parameter 0.001 for NGE and 0.003 for NFT, and k value 100. Seurat was used to determining the number of PCs required by the uniform manifold approximation and projection (UMAP) algorithm. This rate was chosen as 20 for cell-type clustering and 10 for cell-subtype clustering. After clustering, automatic cell type prediction was performed on cell clusters using a cell type enrichment weighted expression algorithm against previously generated Allen brain reference datasets within EWCE under scFlow functions in R (Hodge et al., 2019). Next, the cell annotation text file containing barcodes, cell type, subpopulation type, and group was created.

### 2.7. DGE analyzes of scRNA-seq

The SingleCellExperiment object was divided based on the cell type or subtype. Expression changes in AD samples versus controls were assessed separately in each cell type using a zero-inflation regression analysis with MAST parts of scFlow using a mixed-effects model. The model specification for NGE was zlm(~diagnosis + (1|manifest) + sex + age + PMI + APOE + pc_mito, method = “glmer,” ebayes = F), and for NFT was zlm(~ diagnosis + (1|manifest) + sex + age + PMI + RIN + pc_mito, method = “glmer,” ebayes = F). Expression differences more than twofold and *p.adj* less than 0.05 were the threshold for selecting significant differences.

### 2.8. scRNA-seq alignment

The Whitelist.txt file was provided to UMI tools (v 1.1.2) as a read filtering file to remove adverse droplet reads and PCR duplicates from the trimmed fastq files (Smith et al., 2017). he alignment was performed by STAR with default parameters except for chimSegmentMin = 12, chimJunctionOverhangMin = 10, chimScoreJunctionNonGTAG = −4, BAM Unsorted, and SoftClip Junctions option. The maximum intron length in a single read was set to one million, and the twopassMode option was set. In addition, the bam files were used to compute the counts per million of ENST00000252486 for analyzing *APOE* quantitative trait loci in NGE data.

### 2.9. Detection of splicing sites in scRNA-seq data

First, index and annotator pickle files were built based on the reference genome files with SICILIAN (v 1.0.0; Dehghannasiri et al., 2021). Next, the SICILIAN pipeline was performed on bam files to remove false positive junctions and unbiasedly discover either annotated or unannotated junctions. SpliZ pipeline (v 1.0.0) with default parameters was used in the Nextflow (v 21.04.0) environment to identify notable ASEs between groups in each cell type (Ewels et al., 2020; Olivieri et al., 2022). In order to complete this task, the SICILIAN output and cell annotation text file were combined into a tsv file for use in SpliZ. The grouping_level_1 was set for cell types, while the grouping_level_2 was set for experiment groups. SpliZ is a scalar score that determines the splicing status of each gene in a cell relative to other cells. The *p* value of the deviation between the medians of the SpliZ score of each cell type was measured with the null distribution. *p.adj* less than 0.05 was considered as a selection threshold for significant ASEs. A modified version of SpliZ, called SpliZVD, using eigenvector loadings on the matrix of residues and their SVD decomposition, introduced three sites as the most variable splicing sites in each gene as SpliZsites. The data of significant scZ scores with *p.adj* < 0.05 from post-SpliZ and contributed junctions from post-SICILAN files were added to the SingleCellExpriment object to visualize plots. Pearson and Spearman correlations of ASEs with splicing factor expression changes in each cell were analyzed in R. The splicing factors were selected based on significant differentially expressed genes of the proteins found in GO:0000380, which indicate “alternative mRNA splicing via spliceosome” in the GO database. RPISeq, a family of machine learning classifiers for predicting RNA-protein interactions, was used to predict the interaction between the splicing factor and the transcript based on Random Forest (RF) or Support Vector Machine (SVM) classifiers trained (Muppirala et al., 2011). PRIdictor was utilized to predict the protein binding sites in RNA sequences (Tuvshinjargal et al., 2016).

### 2.10. Detection of APA in scRNA-seq data

In the Nextflow environment, the ReadZS (v 1.0.0) pipeline was utilized to identify APA using bam files and cell annotation text files. The default parameters were employed, except for the numplots, which was set to 40 (Meyer et al., 2022). Also, to avoid calculation time error, the timeout parameter in the withTimeout function in GMM_based_peak_finder.R file of ReadZS was set to 3,000. The peak density plot was visualized with ggplot and Gviz in R.

### 2.11. Gene ontology, pathway enrichment, and protein–protein interaction analyzes

After collecting the important genes from each part of the analysis based on the cell population, the genes were compared with the ontology available in Reactome, KEGG, GO, and Wikipathway databases through the scFlow package in R. By over-representation analyses at the levels of biological process, molecular function, cellular component and biological pathways along with the selection of the most shared terms with *p.adj* less than 0.05, the most likely affected pathways in AD at the cellular level and their network were displayed with important genes through enrich plot (v 1.14.2) and GOplot (v 1.0.2; Walter et al., 2015). Significant ASEs of NGE and NFT, CELF2 and DDX5 as two possible influential splicing factors, and key AD factors include APP, MAPT, PSEN1, PSEN2, BACE1, BACE2, and TREM2were imported into the online Search Tool for the Retrieval of Interacting Genes/Proteins (STRING) database (v12.0; Szklarczyk et al., 2023) for known and predicted protein–protein interaction (PPI). In order to minimize the rate of false positives, PPI confirmed by the experimental study, pathways from curated databases and reported in abstracts of papers published in PubMed were selected. The interactions comprised direct (physical) and indirect (functional) associations between proteins. Also, clustering was performed on the result with an MCL inflation parameter of 3.

## 3. Results

### 3.1. ASEs in bulk RNA-seq data

#### 3.1.1. ASEs in astrocytes

**Figure 2:**
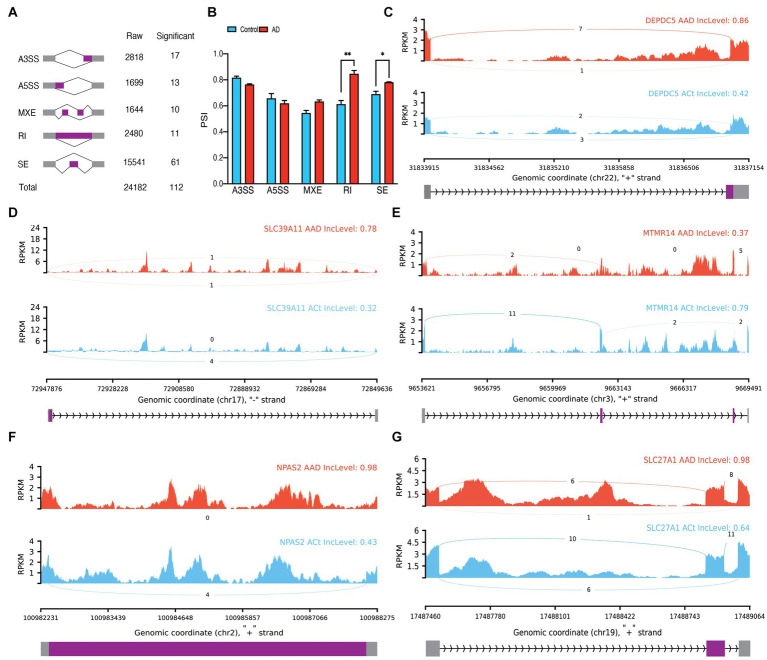
Astrocytes alternative splicing events (ASEs). **(A)** Summary of the raw and significant counts in each ASE. **(B)** The mean of PSI between AD and Control in each ASE. RI and SE are increased in AD with 0.006014 and 0.040209 adjusted *p* values, respectively. **(C)** Detailed sashimi plots for one of the most significant transcripts of A3SS, **(D)** A5SS, **(E)** MXE, **(F)** RI, and **(G)** SE alternative splicing event. Alternative 3′ splice site (A3SS), alternative 5′ splice site (A5SS), skipped exon (SE), retained intron (RI), and mutually exclusive exons (MXE). **(C,D)** In each graph, the AD group is shown at the top (red), the control group is at the bottom (blue), the edited exons are in the middle (purple), the fixed exons are at the sides (gray), and the introns are shown with arrowed lines that indicate the direction of the strand.

In bulk data, we observed a total of 24,182 ASEs from astrocytes and identified 112 significant ASEs (Figure 2A; Supplementary Table S2). Over half of these events were related to SE. Analyzes of the difference between the percent spliced in (PSI) averages of the groups in each type of event showed a significant increase in the average PSI in AD compared to the control in RI (*p* = 0.006014) and SE (*p* = 0.040209) events, indicating an increase in intron conservation and the involvement of alternating exons in the structure of transcribed RNA in AD (Figure 2B). Notable ASEs include an increase in the upstream length of exon 26 of *DEPDC5* in AD (P.ADJ = 6.47E-6), an increase in the downstream length of exon 3 of *SLC39A11* in AD (*p.adj* = 6.38E-5), MXE of *MTMR14* (*p.adj* = 1.97E-6), RI of intron 16 of *NPAS2* (*p.adj* = 6.38E-9), SE of exon seven of *RUFY1* (*p.adj* = 7.55E-7), and SE of exon six of *SLC27A1* (*p.adj* = 6.06E-9; Figures 2C–G). Furthermore, principal component analyses (PCA) revealed that the 112 significant ASEs could cluster samples based on AD (Supplementary Figure S1B). We also identified seven significant differentially expressed genes (DEGs) in astrocytes (Supplementary Figure S1C; Supplementary Table S4) and observed significant DTU in 79 genes (Supplementary Table S3), including *HDHD3* (*q* = 3.815E-20) and *DGKA* (*q* = 1.112E-37; Supplementary Figures S1D–G).

#### 3.1.2. ASEs in endothelial

**Figure 3:**
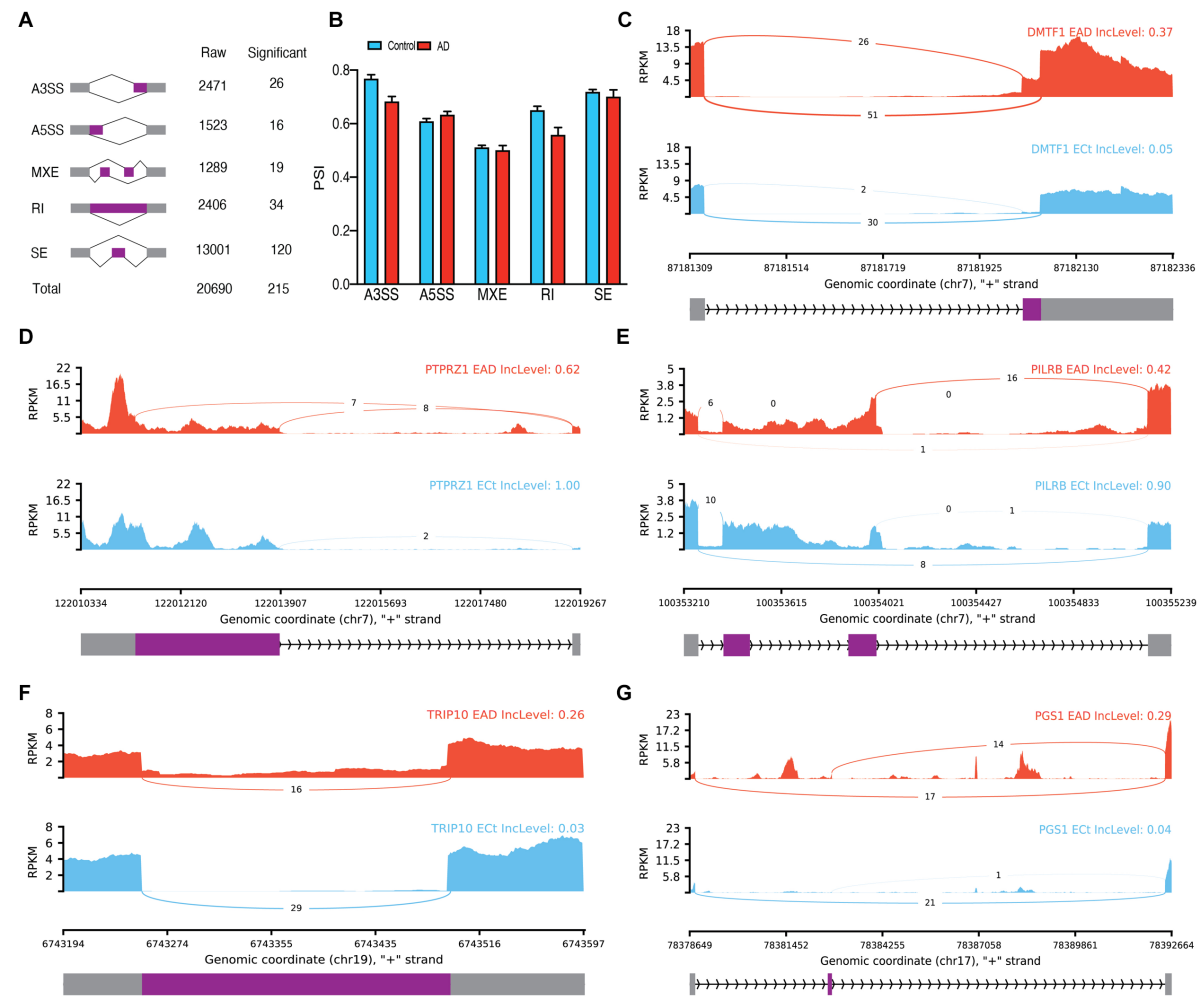
Endothelial ASEs. **(A)** Summary of the raw and significant counts in each ASE. **(B)** The mean of PSI between AD and Control in each ASE. **(C)** Detailed sashimi plots for one of the most significant transcripts of A3SS, **(D)** A5SS, **(E)** MXE, **(F)** RI, and **(G)** SE alternative splicing event. Alternative 3′ splice site (A3SS), alternative 5′ splice site (A5SS), skipped exon (SE), retained intron (RI), and mutually exclusive exons (MXE). **(C,D)** In each graph, the AD group is shown at the top (red), the control group is at the bottom (blue), the edited exons are in the middle (purple), the fixed exons are at the sides (gray), and the introns are shown with arrowed lines that indicate the direction of the strand.

In bulk data from endothelial cells, a total of 20,690 ASEs were identified, out of which 215 were determined to be significant ASEs, primarily SEs (Figure 3A; Supplementary Table S2). No significant differences were observed between the PSI means of the groups in any event (Figure 3B). Notable ASEs included A3SS of exon 10 of *DMTF1*(*p.adj* = 5.95E-11), A5SS of exon 12 of *PTPRZ1* (*p.adj* = 6.36E-5), MXE of *PILRB* (*p.adj* = 0.000013), increasing RI of intron 5 of *TRIP10* in AD (*p.adj* = 1.56E-9), and SE of exon 2 of *PGS1* (*p.adj* = 5.98E-10; Figures 3C–G). Based on the PCA analysis of significant ASEs, two distinct clusters were observed based on AD (Supplementary Figure S2B). Additionally, 400 significant DEG were identified in endothelial cells (Supplementary Figure S2C; Supplementary Table S4), along with 121 significant DTUs (Supplementary Table S3) such as *ARHGEF6* (*q* = 5.94E-9) and *DGKA* (*q* = 2.015E-6; Supplementary Figures S2D–G).

#### 3.1.3. ASEs in microglia

**Figure 4:**
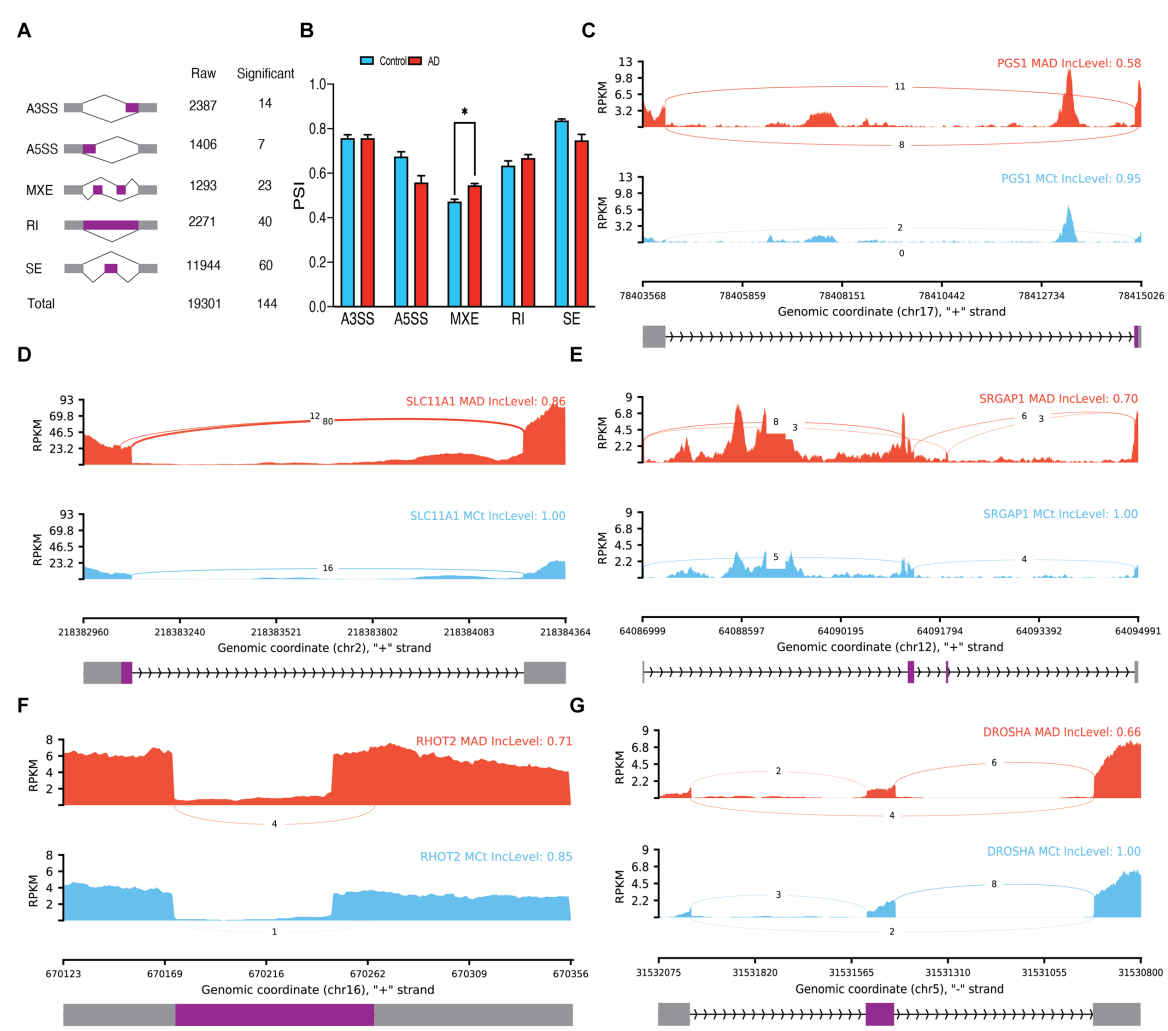
Microglia ASEs. **(A)** Summary of the raw and significant counts in each ASE. **(B)** The mean of PSI between AD and Control in each ASE. MXE is increased in AD with 0.013336 adjusted *p* value. **(C)** Detailed sashimi plots for one of the most significant transcripts of A3SS, **(D)** A5SS, **(E)** MXE, **(F)** RI, and **(G)** SE alternative splicing event. Alternative 3′ splice site (A3SS), alternative 5′ splice site (A5SS), skipped exon (SE), retained intron (RI), and mutually exclusive exons (MXE). **(C,D)** In each graph, the AD group is shown at the top (red), the control group is at the bottom (blue), the edited exons are in the middle (purple), the fixed exons are at the sides (gray), and the introns are shown with arrowed lines that indicate the direction of the strand.

In microglial cells of bulk data, 19,301 ASEs were observed, out of which 144 ASEs were identified as significant (Figure 4A; Supplementary Table S2). The analysis of the difference between the PSI averages of the groups in each type of event revealed a significant increase in the average PSI in AD compared to the control group in MXE events (*p* = 0.013336; Figure 4B). Notably, some of the significant ASEs identified include A3SS of exon 6 of *PGS1* (*p.adj* = 0.000867), A5SS of exon 2 of *SLC11A1* (*p.adj* = 0.00028), MXE of exon 12 of *SRGAP1* (*p.adj* = 7.76E-7), RI of intron 4 of *TJAP1* (*p.adj* = 0.00086), and SE of exon 2 of *DROSHA* (*p.adj* = 0.00022; Figures 4C–G). The PCA analysis revealed that significant ASEs could distinguish AD from the control group (Supplementary Figure S3B). Additionally, 93 significant DEGs were identified in microglia (Supplementary Figure S3C). Moreover, 303 significant DTUs in microglial cells were detected (Supplementary Table S3), which include *RBM38* (*q* = 1.441E-4) and *PARP1* (*q* = 1.084E-5; Supplementary Figures S3D–G).

#### 3.1.4. ASEs in neurons

Out of the 22,522 ASEs observed in neurons, only 15 of them were identified as significant, including 12 SE events, two RI events, and one A5SS event. These events were not sufficient to perform PCA. However, 62 significant DTUs in neurons were detected, as shown in (Supplementary Table S3). Moreover, no DEGs were found for neurons.

### 3.2. ASEs in NGE scRNA-seq data

**Figure 5:**
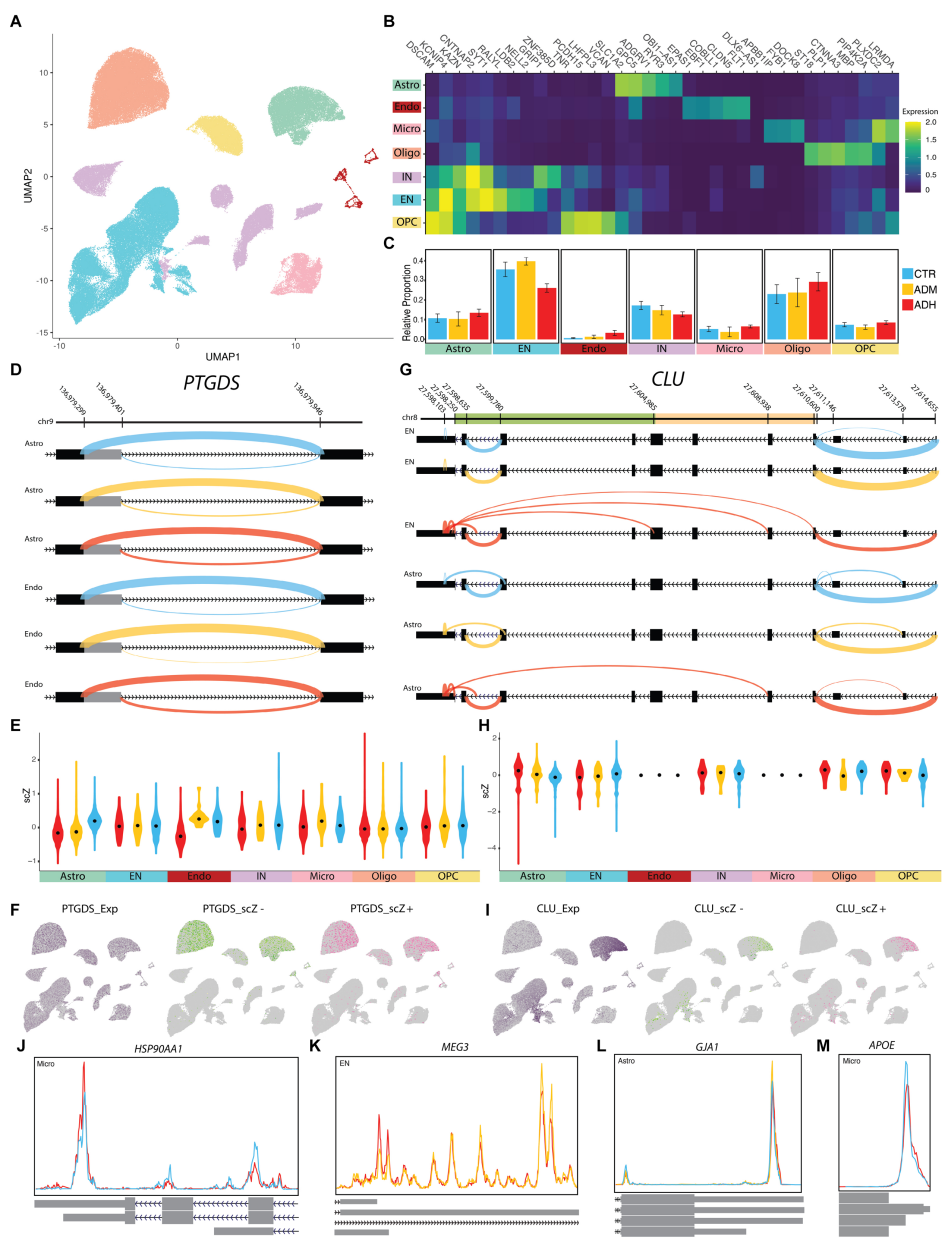
ASEs of NGE data. **(A)** UMAP plot showing the six major cell types of PFC. **(B)** Heatmap of each cell type’s average scaled expression of top enriched genes. **(C)** Proportions of each cell type found in ADH, ADM, and CTR of PFC. **(D)** The sashimi plots of *PTGDS* transcripts show an A5SS event with differential scZ median in astrocytes and endothelial cells. **(E,H)** The violin plots show the distribution of scZ scores in each population by the group. Black dots represent the median. **(F,I)** The UMAP plots demonstrate cells with the gene expression in purple, cells with negative scZ in green, and positive in pink. **(G)** The sashimi plots of *CLU* transcripts show abnormal splicing in excitatory neurons (EN) and several ASEs with differential scZ median in EN and astrocyte cells; CLU beta domain (light orange) and alpha domain (green). **(J–M)** The Peak plots present differential polyadenylation sites **(J)** *HSP90AA1* in microglia, **(K)** *MEG3* in EN, **(L)** *GJA1* in astrocyte, and **(M)** *APOE* in microglia, which is correlated with rs429358-C (Supplementary Figure S6). Cyan lines indicate CTR, yellow lines indicate ADM, and red lines indicate ADH. CTR, control; ADM, Alzheimer’s disease with moderate severity; ADH, Alzheimer’s disease with high severity.

In NGE data, 34 distinct clusters were detected by the clustering algorithm in 7 major cell groups, including astrocytes, endothelial cells, microglia, oligodendrocytes, excitatory neurons, inhibitory neurons, and oligodendrocyte progenitor cells (OPC; Figures 5A,B; Supplementary Figure S4). The algorithm was based on the co-expression of genes between close cells and the main components of the uniform manifold approximation and projection (UMAP) dimension reduction algorithm. Although the relative population of neurons appeared to decrease with increasing disease severity, and the relative population of glial cells and endothelial cells appeared to increase with increasing disease severity, these observations were not statistically significant (*p* > 0.05; Figure 5C). Furthermore, there was no significant correlation between the postmortem interval (PMI) and the number of genes and transcripts in each sample (Supplementary Figures S5A,B). The metavariables, including donor, age, gender, PMI, and *APOE* gene alleles, were relatively uniformly distributed among the clusters (Supplementary Figures S5C–I).

A total of 3,444 cases of ASEs were observed across all three groups. Of these, only 130 were deemed significantly altered. Specifically, 1,856 ASEs were detected between ADM and control, with 57 being deemed significant. Between ADH and control, 3,040 ASEs were identified, with 105 being significantly changed. Lastly, 1,604 ASEs were observed between ADM and ADH, of which only 60 were deemed significant (Supplementary Table S5). Some notable examples of changes are outlined below. Despite *PTGDS* being relatively similarly expressed across all cell types, alternative splicing changes of *PTGDS* transcripts were significantly observed in astrocyte (*p.adj* < 1E-20) and endothelial populations (*p.adj* = 0.00132), affected by A5SS upstream of exon three (Figures 5D–F). Specifically, in astrocytes, the longer exon was more frequently observed in both ADH and ADM, while the longer exon in endothelial cells was increased only in ADH. ASEs of *CLU* transcripts were significantly observed in astrocyte (*p.adj* = 4.21E-14) and EN populations (*p.adj* = 0.00012), with regions close to the 3′ end of its transcripts in both astrocytes and ENs representing a model of disrupted alternative splicing (Figures 5G–I). Some *CLU* transcripts with incomplete and short open reading frames (ORF) were observed. This disruption in ADM and control astrocytes was seen in the form of abnormal junctions of the internal regions of exon 7, which are close to the 5′ splicing site, with the internal regions of the 3′UTR. Another case of these abnormal junctions was found in the same location of the 3′UTR internal regions but with different locations, including intronic regions in ADM astrocytes. This splicing disruption is more evident in ADH ENs to the extent that some transcripts lack the coding region or the whole alpha domain. In addition, it seems that the 3′UTR was shortened in both ENs and astrocytes as the severity of the disease increased.

The study also revealed significant APA in the transcripts of 26 genes. Notably, in microglia, *HSP90AA1* transcripts with three additional coding exons at the 3′ end were increased in ADH compared to the control (Figure 5J). *MEG3*, a lncRNA, showed decreased length at the 3′ end in ENs with increasing disease severity (Figure 5K). In astrocytes, longer transcripts of *GJA1* were increased in ADM but decreased in ADH compared to the control (Figure 5L). In microglia, the 3′ end of *APOE* transcripts were decreased in ADH compared to the control (Figure 5M). However, allelic association analyses of *APOE* rs429358 polymorphism with increased splicing of the longer ENST00000252486 transcript in microglia (*p* = 0.8624) and astrocytes (*p* = 0.1594) were not confirmed (Supplementary Figure S6).

Specific distribution patterns in some sub-population areas were observed in the UMAP graphs of scZ scores, prompting a more detailed investigation at the cluster level of each population (Figures 5F,I). The investigation revealed that alternative splicing variation of some genes in astrocytes and ENs occurred only in certain subtype clusters. In astrocyte clusters 1 and 2, which express synaptic support genes, ASEs in *FTL*, *CLU*, and *MT3* genes were observed, similar to cluster 6. Meanwhile, *PTGDS* showed ASEs in almost all astrocyte populations (Supplementary Figures S7A–D). In neuronal clusters, the EN-L5-6 subtype showed expression profiles in some cells more similar to other subpopulations than their own. EN-L5-6 comprised clusters 3, 11, 13, 15, and 17, with relative ratios of ADH and ADM being higher in clusters 15 and 17, which had the highest *SYT1* ASEs compared to other EN-L5-6 clusters. Conversely, the control group showed higher relative ratios in cluster 3 than the other groups (Supplementary Figures S7E–I). Therefore, to understand the effects of tau on alternative splicing in PFC neuronal subtypes, we assessed ASEs between neurons with and without tau pathology.

### 3.3. ASEs in NFT scRNA-seq data

**Figure 6:**
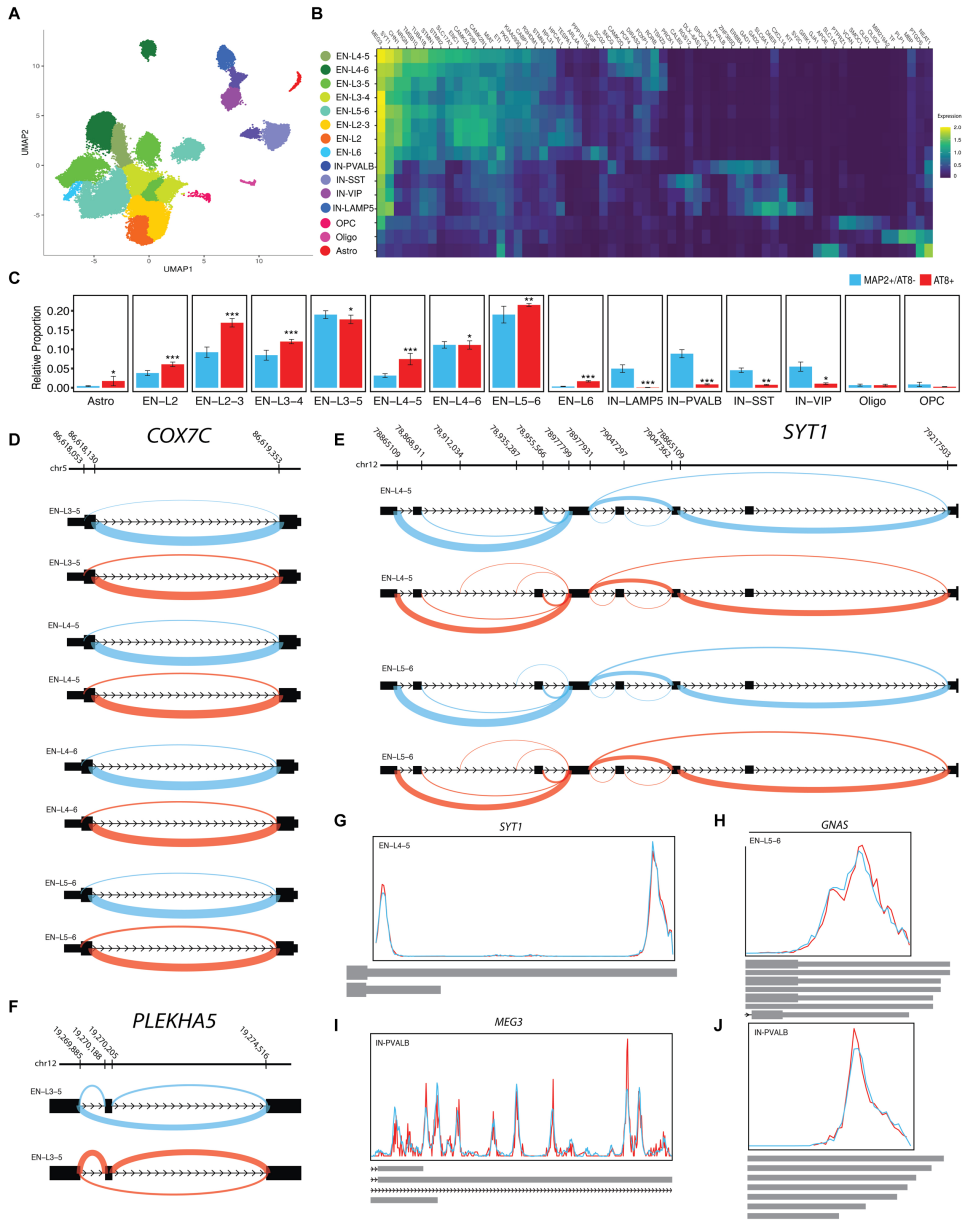
ASEs of NFT data. **(A)** UMAP plot showing the neuron subtypes of PFC. **(B)** Heatmap of the average scaled expression of top enriched genes for each subtype. **(C)** Each subtype was reported in ADH, ADM, and CTR of PFC. **(D)** The sashimi plots of *COX7C* transcripts show the A5SS event with differential scZ median in EN-L3-5, EN-L4-5, EN-L4-6, and EN-L5-6. **(E)** The sashimi plots of *SYT1* transcripts show abnormal splicing in excitatory neuron subtypes and several ASEs with differential scZ median in EN-L4-5 and EN-L5-6. **(F)** The sashimi plots of *PLEKHA5* transcripts show SE event with differential scZ median in EN-L3-5. **(G–J)** The Peak plots present differential polyadenylation sites **(G)** *SYT1* in EN-L4-5, **(H)** *GNAS* in EN-L5-6, and **(I,J)** *MEG3* in IN-PVALB with multiple sites. Cyan lines indicate MAP2+/AT8−, and red lines indicate AT8+. Indicated *p* value as less than 0.05, 0.01, and 0.001 illustrated with one, two, and three stars, respectively.

The clustering algorithm detected 25 distinct clusters in 15 major cell groups, including eight EN cell lines classified based on their cerebral cortex location, four IN cell lines, namely IN-PVALB, IN-SST, IN-LAMP5, and IN-VIP, and three non-neuronal cell types, including oligodendrocytes and OPC in NFT data (Figures 6A,B; Supplementary Figure S8). In AD, tau pathology affects EN populations but not IN populations (Figure 6C). The PMI showed no significant correlation with the number of genes and transcripts in each sample (Supplementary Figures S9A,B). Furthermore, metavariables, such as donor, age, gender, PMI, and RNA integrity number (RIN), were relatively uniformly distributed among the clusters (Supplementary Figures S9C–I).

A total of 4,704 cases of ASEs were observed between neurons with and without NFT, out of which only 256 ASEs were significantly changed (Supplementary Table S6). Additionally, significant APA was detected in the transcripts of 18 genes. Some of the most noteworthy changes are described below. In EN-L5-6 (*p.adj* < 1E-20), EN-L4-6 (*p.adj* = 4.26E-13), EN-L4-5 (*p.adj* = 1.37E-12), and EN-L3-5 (*p.adj* = 3.10E-9) populations, significant alternative splicing changes of *COX7C* transcripts were observed, wherein the upstream of exon one was subjected to A3SS. Consequently, the expression of noncoding transcript ENST00000511472.5 was increased in neurons with NFT (Figure 6D). ASE of *SYT1* transcripts was significantly observed in EN-L4-5 (*p.adj* = 0.00253) and EN-L5-6 (*p.adj* = 0.00431) populations, wherein a model of alternative promoter was observed in its 5′UTR (Figure 6E). There was a significant increase in *PLEKHA5* transcripts in the EN-L3-5 population, with exon 10 experiencing SE. This led to an increase in the expression of the ENST00000429027.7 transcript, which includes exon 10, in neurons with NFT (Figure 6F).

Regarding APAs, we observed that transcripts of *SYT1* in EN-L4-6 with shorter 3’UTR were slightly increased in neurons with NFT (Figure 6G). In addition, an increase in the length of the 3′ end of GNAS transcripts was observed in EN-L5-6 with NFT (Figure 6H). In IN-PVALB with NFT, *MEG3* transcripts were elongated at the 3′ end with longer exons, such as ENST00000522771.9, which terminate before the range of chr14:100845000_100850000 (Figure 6I). However, the length was reduced at the end of 3′ transcripts with the transcription termination region in the range of chr14:100860000_100865000 (Figure 6J).

### 3.4. DGE of single cell data

Several DEGs were identified in the astrocyte and EN populations of the NGE data (Supplementary Figure S10; Supplementary Table S7). When comparing ADH to control, most DEGs were down-expressed in astrocytes, while they were up-expressed in neurons. No significant expression changes were detected in the comparison of ADM endothelial cells with control and in OPCs. DGE analyses of different astrocyte clusters demonstrated significant changes depending on the cluster. However, genes such as *GFAP* were increased in all comparisons with the control condition, with a higher increase observed in ADH than in ADM. Additionally, DTU genes such as *CLU*, *APOE*, *HSP90AA1*, and *AHI1* were also identified among the DGE results. Most DEGs were observed between neurons with and without NFT in ENs and IN-PVALB, while other INs had only a few DEGs (Supplementary Figure S11).

**Figure 7:**
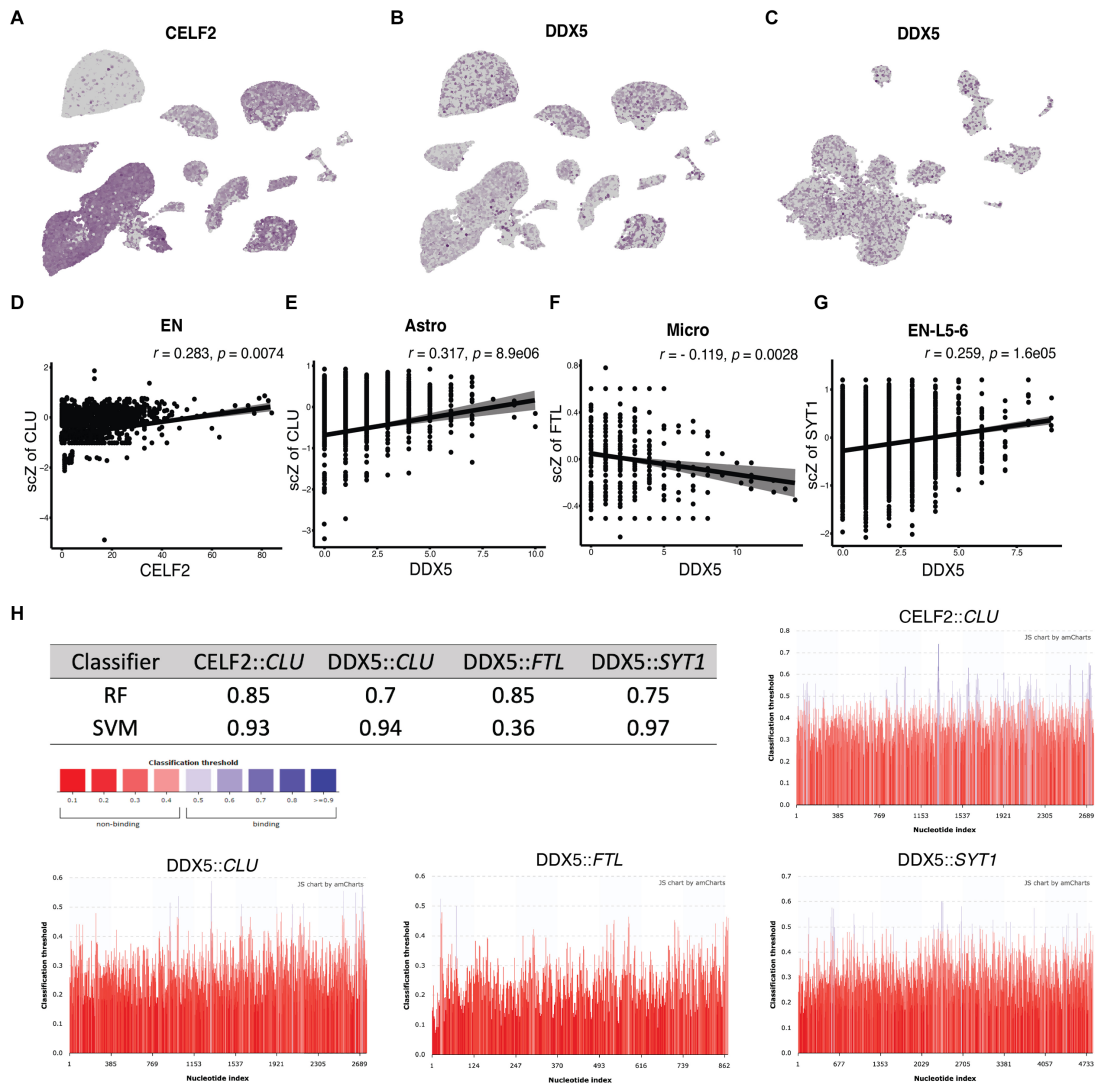
Correlation of *CELF2* and *DDX5* expression with ASEs in single cells. **(A–C)** The UMAP plots show the expression pattern of **(A)** *CELF2* in NGE data, **(B)** *DDX5* in NGE data, and **(C)** *DDX5* in NFT data. **(D–G)** The scatter plots indicate the correlation between the splicing factor expression level on the horizontal axis and the scZ score on the vertical axis in each cell. *r* and *p* values are demonstrated on top of each plot for the Pearson correlation coefficient. **(D)** Correlation between *CELF2* expression and ScZ score of CLU in EN. **(E)** Correlation between *DDX5* expression and ScZ score of CLU in astrocyte. **(F)** Correlation between *DDX5* expression and ScZ score of FTL in microglia. **(G)** Correlation between *DDX5* expression and ScZ score of SYT1 in EN-L5-6. **(H)** The table shows the classifier prediction values of the binding probability between the splicing factor and the transcript with the RPISeq predictor. The colored bar chart shows prediction scores at every position of the sequence. Scores higher than 0.5 indicates the probable binding site and are colored blue. RF, random forest; SVM, support vector machine.

Furthermore, our analysis identified DGEs associated with alternative splicing regulation. Specifically, *CELF2* exhibited a 6.3-fold decrease in expression in ADM astrocytes compared to control (*p.adj* = 1.64E-9), a 4.2-fold decrease in ADH astrocytes compared to ADM (*p.adj* = 2.37E-15), a 9.8-fold decrease in ADM ENs compared to control (*p.adj* = 1.2E-5), and an 11.3-fold decrease in ADH ENs compared to ADM (*p.adj* = 3.15e-8; Figure 7A). Additionally, *DDX5* showed an 8.9-fold decrease in expression in ADH astrocytes compared to ADM (*p.adj* = 1.85E-17), a 2.3-fold decrease in ADH ENs compared to control (*p.adj* = 5.42E-18), a 41.6-fold decrease in ADH microglia compared to ADM (*p.adj* = 1.48E-10), and a 2.6-fold decrease in expression in EN-L5-6 with tau (*p.adj* = 0.00045; Figures 7B,C). Further correlation analyses revealed a correlation between *CELF2* expression and scZ score distribution. Cells expressing lower levels of *CELF2* exhibited more splicing disruptions in *CLU* transcripts in ENs (Figure 7D). Moreover, a direct correlation was observed between decreased expression of *DDX5* and increased disrupted transcripts in *CLU* variants in astrocytes, *FTL* ASEs in microglia, and *SYT1* in EN-L5-6 (Figures 7E–G). Algorithms predicting protein binding to RNA predicted that CELF2 and DDX5 bind to *CLU* and DDX5 binds to *SYT1*, but not in the case of DDX5 binding to *FTL* (Figure 7H). In the case of *CLU*, it is predicted that CELF2 and DDX5 binding sites are located in the middle and end of 3′ transcripts, in the vicinity of the acceptor and donor regions of misplaced connections.

### 3.5. Impacted pathways in AD

**Figure 8:**
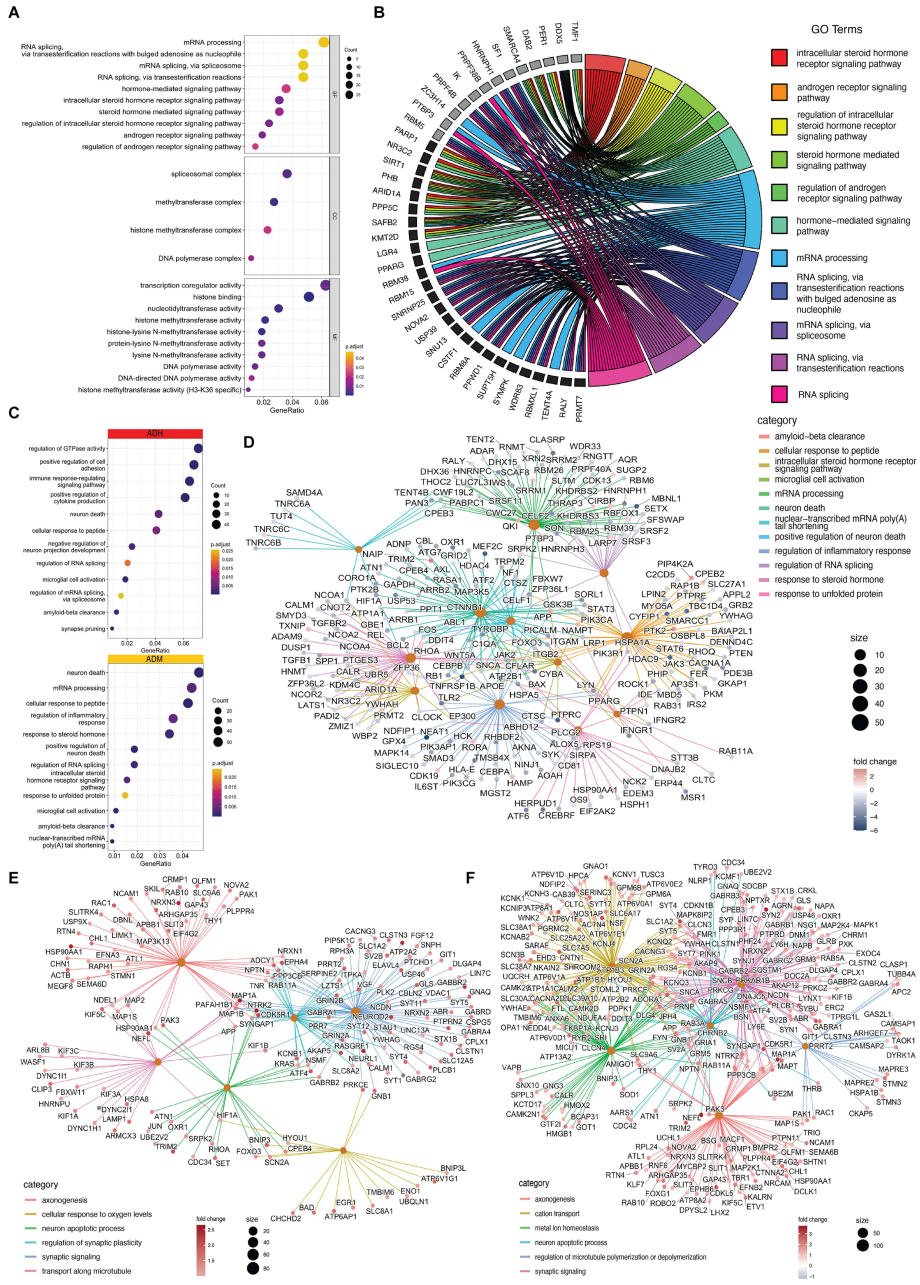
Impacted pathways of PFC cells in AD. **(A)** The dot plots show that GO terms significantly changed in microglia. **(B)** the chord plot indicates that steroid hormone signaling and RNA splicing impact the most significant ASE (black) and DTU (grey). **(C)** Biological processes are changed in ADH (red) and ADM (yellow) microglia. **(D)** Cnetplot shows the relation of ASE, APA, and DGE-involved genes with GO terms in ADM microglia. **(E,F)** Cnet plots present the relation of ASE, APA, and DGE-involved genes with GO terms in **(E)** EN-L5-6 and **(F)** EN-L4-5.

Most cell types in each dataset exhibited expression changes related to AD pathophysiology, with microglia and EN showing splicing and expression changes in RNA processing and splicing pathways. The bulk sequencing data suggested that splicing changes in microglia targeted steroid hormone signaling pathways and genes involved in RNA processing, including splicing themselves (Figure 8A). Cellular components such as spliceosomal complexes, methyltransferase, replication, and related chemical reactions were also targeted. The affected pathways identified in the single-cell sequencing data of microglia in ADM were similar to those observed in the bulk sequencing data, with pathways affecting the response to steroid hormones and RNA processing, as well as those impacting neuronal death, Aβ clearance and inflammatory responses, which are hallmarks of AD (Figures 8B–D). In EN-L5-6 neurons with NFT, the axonogenesis pathway network, response to oxygen levels, cell death, synaptic signaling, and synaptic plasticity were targeted (Figure 8E). In the population of EN-L4-5 neurons with NFT, a more extensive network of genes was also present, including metal ions targeted in synaptic signaling to the cytoskeleton and homeostasis (Figure 8F).

**Figure 9:**
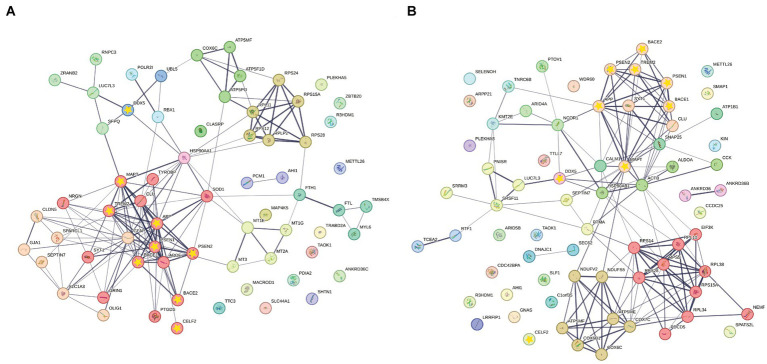
STRING Protein–protein interaction (PPI) analyses. **(A)** PPI network connectivity for proteins identified as differentially spliced in NGE data represented with normal bubbles and key etiological proteins of AD or DDX5 and CELF2 represented with a yellow star inside bubbles. The network contains 11 clusters and 66 nodes with 157 edges (vs. 74 expected edges); clustering coefficient 0.481; enrichment value of *p* < 1.0e-16; average node degree 4.76. **(B)** PPI network connectivity for proteins identified as differentially spliced in NFT data represented with normal bubbles and key etiological proteins of AD or DDX5 and CELF2 represented with a yellow star inside bubbles. The network contains 12 clusters and 71 nodes with 157 edges (vs. 94 expected edges); clustering coefficient 0.475; enrichment value of p 1.53e-09; average node degree 4.42. Edge line thickness indicates the strength of data support. Each cluster has the same color bubbles.

Furthermore, STRING protein–protein interaction analysis was performed on significant ASEs of NGE and NFT with the addition of CELF2 and DDX5 as two possible influential splicing factors, and key AD factors include APP, MAPT, PSEN1, PSEN2, BACE1, BACE2, and TREM2 to discover whether the identified differentially spliced transcripts have potential known functional interactions with AD factors (Figures 9A,B). Notably, the first biggest cluster of NGE data and the second biggest cluster of NFT data contributed to AD pathogenesis and have a role in clearing amyloid and tau in the brain. Also, significant interactions were observed in other biological pathways involved in neurodegeneration, such as ion homeostasis, glial cell activation in immune response, regulation of reactive oxygen species metabolic process, cytoplasmic translation, and RNA processing. Each cluster has the same color bubbles.

## 4. Discussion

In recent years, advancements in molecular techniques such as GWAS and RNA-seq have facilitated the identification of potent molecules associated with the pathology of AD, which were previously overlooked (Penney et al., 2020). However, these studies were frequently confined to analyzing tissues rather than individual cells and prioritized gene expression over transcript expression, representing a more functional level than gene expression. This study addresses these limitations by examining cell-type-specific transcripts alterations in AD, achieving a more comprehensive analysis of AD transcriptomics data than previous studies. To the best of our knowledge, only two studies have attempted to identify DTUs in Alzheimer’s disease AD brain and AD model based on cell types. Marques-Coelho et al. indirectly assigned genes to unique cell types in the medial temporal gyrus region of AD brains and compared DEGs/DTUs from bulk RNAseq to unique cell populations from previously published scRNAseq data (Marques-Coelho et al., 2021). In contrast, our study directly analyzed scRNAseq reads in the PFC of the AD brain. In a separate investigation, Weller et al. identified DTUs in scRNAseq data from the hippocampus of APOE null mutant mice using Sierra (Weller et al., 2022), which uses pseudobulk analyses to detect DTUs and peak calling to detect APA (Patrick et al., 2020). Our method employed SpliZ scalar quantification to measure true exon junctions between cell types and detected APA using at least two peaks in human postmortem data. The present study highlights the cell type and subtype-specific alterations in splicing patterns of certain genes in AD, even in the absence of expression changes. Notably, an increase in ASEs is observed with disease progression. We used an annotation-free approach to scRNAseq data analysis to identify novel splicing ASEs and disruptions. Collectively, our findings offer a detailed account of cell type-specific modifications in gene expression in the AD brain and imply that alternative splicing may play a significant role in the pathological progression of the disease.

**Figure 10:**
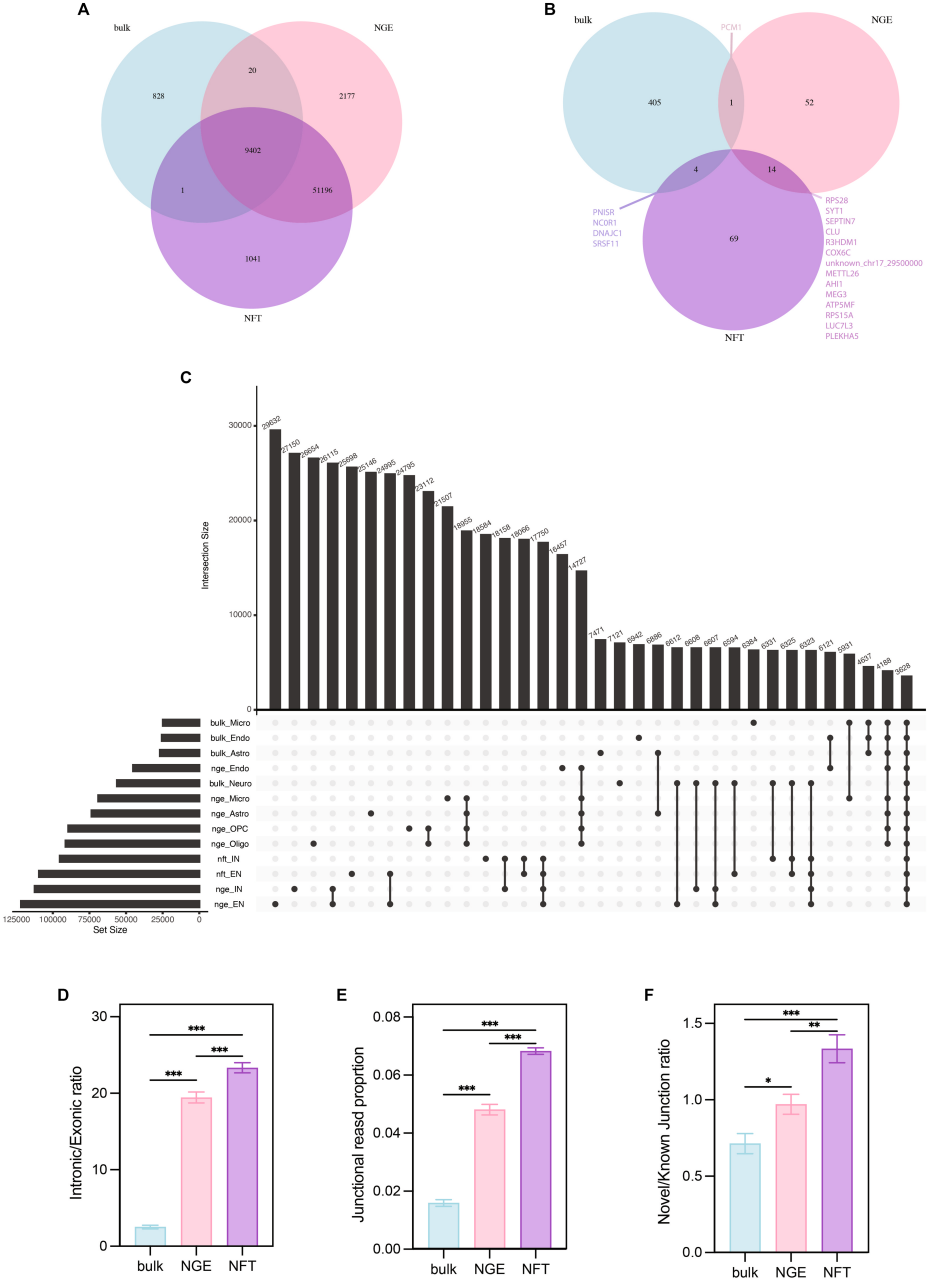
Comparison among bulk, NGE, and NFT. **(A)** Venn diagram indicates that numbers of total ASEs identify in each data. **(B)** Venn diagram illustrates the numbers of total significant ASEs in each study. **(C)** The upset plot compares total annotated ASEs based on the cell type and study. **(D)** Comparison intronic/exonic ratio, **(E)** junctional reads proportion, and **(F)** novel/known junction ratio among studies by ANOVA *post hoc* Tukey. *p* < 0.05 (*), *p* < 0.01 (**), and *p* < 0.001 (***).

In order to cover the shortcomings of each technique in this study, the data of three different RNA sequencing approaches, including bulk RNA-seq, single nuclei RNA-seq, and single soma RNA-seq, were used to identify ACEs in AD. While bulk sequencing provides full-length coverage of transcripts in contrast to 10x single-cell sequencing, it is biased in favor of more populated subtypes. snRNA-seq is a widely used method to determine brain cell type complexity and is used to construct a comprehensive human brain cell atlas, although it relies on the nuclear mRNA pool (Kim et al., 2023; Pavan et al., 2023). While bulk RNA sequencing data shows a strong correlation between nuclei and whole-cell samples in differential expression analysis, at the single-cell or single-nucleus levels, cell-to-cell or nucleus-to-nucleus correlations decrease and replicate variations become larger than the bulk samples (Kim et al., 2023). Recent studies report that the single-nucleus RNA sequencing technique is biased towards genes with longer sequence lengths and roughly >10 exons, whereas the single-cell RNA sequencing technique captures shorter genes more efficiently (Gupta et al., 2022; Pavan et al., 2023). Moreover, Characterization of the nuclear and cytosolic transcriptomes in human brain tissue reveals that transcripts encoding nuclear-encoded mitochondrial proteins are significantly enriched in the cytosol, while lncRNAs significantly enriched in the nucleus (Zaghlool et al., 2021). Therefore, these studies suggest that examining single-cell sequencing data alongside bulk sequencing data is better to gain a more detailed insight. Our analysis showed that most of the known junctions were identified in common across all three studies, bulk, NGE, and NFT (Figure 10A). There were 51,196 intersected ASEs between NGE and NFT, most including unannotated junctions. After we applied a threshold of 0.05 on the *p.adj* values, the number of genes with significant splicing changes in NGE and NFT was less than the bulk data (Figure 10B). There were more intersected genes with significant splicing alteration between NFT and NGE rather than bulk with NFT or NGE. The most intersected differential annotated junctions were observed between NFT and NGE neurons (Figure 10C). In every study, most differential annotated junctions were observed in neurons rather than the other cell types. However, more significant ASEs were observed in NFT and NGE neurons data than in the bulk neurons data. This highlights the advantage of single-cell data, which ignores the splicing variation between different cell subtypes by comparing diagnoses in a given subtype. The intronic/exonic ratio, junctional reads proportion of the total reads, and novel/known junction ratio was higher in NFT than NGE and higher in NGE than bulk data (Figures 10D–F).

This paper reports several cases of SE closely related to AD pathology, while the effects of others remain unclear. One such case is *SLC27A1*, expressed explicitly in astrocytic clusters four and six and involved in transporting fatty acids from the BBB to the brain (Ochiai et al., 2017). SLC27A1 plays a vital role in brain health by absorbing endogenous neuroprotective factors such as docosahexaenoic acid and biotin from the blood. However, an *in vitro* study shows that Aβ inhibits the uptake of docosahexaenoic acid by SLC27A1 from the environment (Ochiai et al., 2019). Moreover, the absorption of substances by SLC27A1 is related to the presence of insulin in the environment (Ochiai et al., 2017). Our findings suggest an SE in the coding region of the cytoplasmic regulatory subunit of AMP-binding protein, which may be related to the insulin signaling pathway. Another case is DROSHA, an RNase enzyme that plays a crucial role in the processing of microRNAs. Our results indicate that transcripts of *DROSHA* are significantly SE in exons two, six, and seven in microglia, resulting in a reduction in the length of the 5′ UTR region in AD and an increase in the presence of exon seven in the structure of DROSHA transcripts in AD microglia. Recent studies have reported different types of *DROSHA* transcripts that show subcellular localization differences, although they do not exhibit apparent functional differences in the processing of microRNAs. Interestingly, this process appears to be cell type-specific, and the presence of exon seven in *DROSHA* mRNA is essential for its cytoplasmic localization (Dai et al., 2016; Link et al., 2016). In addition, new evidence suggests that increased Aβ in postmortem brains and rat brains reduces the presence of DROSHA in the nucleus and increases its presence in the cytoplasm. This phenomenon is associated with the phosphorylation of DROSHA by p38 MAPK in neurons, and the DROSHA phosphorylation site is located near the junction of exon eight to seven (Xu et al., 2021). Another example is *PLEKHA5* transcripts, which show SE that includes an additional exon between exon nine and ten. This SE increases the mRNA structure in EN-L3-5 with NFT, and this region encodes the Pleckstrin homology domain, which regulates plasma membrane and trans-Golgi membrane traffic activities (Sluysmans et al., 2021). Finally, we observed that RI is another type of ASE, significantly increased in astrocytes. For instance, *NPAS2*, which is involved in the circadian rhythm, has a splicing disorder in its transcripts that may be related to sleep disorder in AD (Sharma et al., 2021).

The findings pertaining to astrocytes indicate that alternate splicing in this cell type is closely associated with Alzheimer’s disease (AD). Moreover, certain sub-types exhibit distinct patterns of alternative splicing and expression linked to specific AD pathologies. Notably, astrocytes exhibit significantly impaired splicing of *CLU* transcripts, a gene highly expressed in astrocytes. CLU is an extracellular chaperone that interacts and binds to Aβ, reducing its aggregation and promoting its clearance, suggesting a potential neuroprotective role. However, some studies have suggested that CLU may contribute to the spread of Aβ in the brain and serve as a key mediator in Aβ-induced neurotoxicity. This interaction may depend on the distribution ratio of CLU to Aβ (Foster et al., 2019). Most of the astrocyte-related CLU splicing disruptions observed in this study are related to the alpha domain. Some of the abnormal junctions miss the glycosylation and phosphorylation residues of CLU, which disruption of these structures can lead to a decrease in the polarity of the protein and a suitable substrate for the deposition of insoluble compounds such as Aβ and NFT or Its secretion is prevented. The *CLU* gene is known as the third risk factor for late-onset AD. Studies of allele-specific quantitative loci indicate that *CLU* splicing changes are related to rs9331896 and rs7982 polymorphisms in the intronic region of the *CLU* gene (Raj et al., 2018; He et al., 2022). Another crucial gene in astrocytes is *APOE*, the first risk factor for late-onset AD, which undergoes APA and reduced expression in astrocytes. In humans, there are three common types, ApoE3, ApoE2 and ApoE4, which differ by only one amino acid at position 112 or 158. APOE plays a vital role in Aβ clearance, which ApoE2 has the strongest effect on Aβ clearance, followed by ApoE3 and least of all ApoE4 (Huynh et al., 2017). Studies of allele-specific quantitative expression loci reveal an association between *APOE* ASE and rs429358 polymorphism, coding ApoE4, in the coding region of the 3′ end of the *APOE* gene (He et al., 2022). Although the PCR data related to this polymorphism was available for the samples used in this study, the correlation between the allele and the reduction of the length UTR3’ and the N-terminal coding of APOE was not statistically significant, possibly due to the smaller sample size compared to the previous study. A network of genes involved in metal homeostasis, oxidation, and metabolism, such as *FTL*, *MT1E*, *MT1G*, and *MT3*, is evident in astrocyte clusters related to the nutritional support of neurons and synapses. Disruption in the transcripts related to these genes primarily leads to autophagy disorder, one of the pathological features of AD (Koh and Lee, 2020). Furthermore, a significant decrease in the expression of *DDX5*, an RNA helicase that regulates spliceosome structure and prevents the formation of unwanted RNA loops, was observed in astrocytes. This decrease correlated with an increase in the dispersion of scZ scores of *CLU* transcripts. Additionally, studies suggest that DDX5 increases the access of U1snRNP to the 5′ binding site of MAPT exon ten by structurally changing the stem-loop structure, which prevents the formation of incomplete tau proteins (Kar et al., 2011). Additionally, studies suggest that DDX5 increases the access of U1snRNP to the 5′ binding site of *MAPT* exon ten by structurally changing the stem-loop structure, preventing incomplete tau proteins (Li et al., 2021). PTGDS is a highly abundant protein found in cerebrospinal fluid that has been suggested to act as an endogenous chaperone for Aβ. The expression level of *PTGDS* is related to dementia, with its expression being regulated both directly and indirectly by estradiol (Unno et al., 2015). It has been reported that plasma levels of PTGDS in AD patients are associated with increased levels of inflammatory cytokines and reactive oxygen species (Dharshini et al., 2019). Our study revealed that the A5SS event of *PTGDS* transcripts endothelial and astrocyte cells. UniProt data suggests this event occurs within the coding region of turn between beta sheets with disulfide bonds near the enzyme’s active site, potentially impacting the enzyme’s regulatory activities. A recent NMR study has shown that PTGDS can inhibit primary and secondary nucleation of Aβ40 by interacting with both monomers and the surface of fibrils, reducing the final fibril content (Kannaian et al., 2019). The proposed binding region of PTGDS with Aβ is adjacent to the turn affected by the A5SS event in *PTGDS*.

Our analysis revealed that genes contributed to RNA splicing and the steroid hormone signaling pathways in microglia undergoing DTU and DGE. Studies suggest that the loss of ovarian hormones during menopause may increase the risk of AD in women, as androgens play a crucial role in various brain functions, such as neurotransmission, neurodevelopment, survival, protection against oxidative stress, reduction of Aβ peptide levels, and reduced tau hyperphosphorylation (Breijyeh and Karaman, 2020). Furthermore, our findings showed a correlation between the splicing changes of FTL transcripts and *DDX5* expression. In microglia, FTL has increased expression and alternative splicing of exon 3, despite only one FTL transcript in the reference genome and the skipping of exon three observed in ADH being considered abnormal. Exon three encodes FTL alpha helices, and mutations in this region have been linked to Parkinsonian symptoms, ataxia, and mild non-progressive cognitive impairment (Maciel et al., 2005). Our analysis also revealed several alternative APAs in microglia. Moreover, we observed expression changes in factors involved in shortening the poly A tail of mRNA molecules synthesized in the nucleus of microglia. While *APOE* splicing changes were also observed in microglia, we did not statistically confirm its relationship with rs429358. HSP90AA1, an intracellular chaperone that corrects misfolded proteins and prevents their disruptive aggregation, underwent 3′ end elongation in ADH. Studies suggest that this difference in elongation may be due to alternative promoter recognition by different transcription factors in combination with RNA polymerase rather than APA. An increase in this transcript’s 3′ end length is associated with factors such as viral infection, inflammation, cell death, and increased glucose concentration(Zuehlke et al., 2015; Bohush et al., 2019). Increased expression of *HSP90AA1* was also observed in microglia and EN in other studies (Bohush et al., 2019).

In contrast to the bulk sequencing data, which showed limited cases of ASEs and DTU and lacked any DGE in neuronal types, single-cell sequencing data revealed multiple cases of expression and transcriptome changes. This may be due to the wide transcriptome difference between EN and IN or may be due to an error in the neuron collection protocol. The events identified in neurons using single-cell sequencing data appear to be repeated in the bulk sequencing data but are not statistically significant. Increasing the number of donors for neuron lineages may improve this issue. Nonetheless, important genes such as *TSPAN14* are observed in the DTU of the bulk data. Studies on quantitative allele-specific expression loci methylation support the hypothesis that increased brain expression of *TSPAN14* is linked to an increased risk of AD (Bellenguez et al., 2022). *CLU* is an example of widespread dysregulation of ENs transcripts, and this disruption seems to enhance with disease severity. This irregularity increased correlated with the decrease of *CELF2* and *DDX5* expression. CELF2, also called ETR3, has been identified as a novel risk factor associated with AD, particularly in individuals carrying the high-risk *APOE* ε4 allele, and it has also shown significant association with AD independent of *APOE* ε4, indicating its potential as a valuable therapeutic target for addressing underlying genetic causes of the disease (Tran, 2023). Prior research has demonstrated that the alternative splicing of exon three in *TREM2*, a genetic risk factor for AD, is regulated by two paralogous RNA-binding proteins, CELF1 and CELF2, with CELF2 being implicated in the reduction of full-length TREM2 protein expression through exon three skipping and nonsense-mediated mRNA decay, which effects on microglial responses to the Aβ aggregation (Yanaizu et al., 2020). Also, CELF2 is involved in influencing various transcripts, such as different exons of *MAPT* and *NMDAR1* exon five, in the mis-splicing event in myotonic dystrophy type 1, particularly in reducing the inclusion of *MAPT* exon 10, indicating its role in alternative splicing regulation and potential nuclear and cytoplasmic functions in the brain (Liu et al., 2021). To understand the specific relationship between CELF2 and tau splicing in AD, further studies are needed to investigate the interactions between CELF2 and the tau gene in AD-affected brain tissues.

In addition to protein-coding genes, our study identified changes in transcripts of lncRNAs such as *MEG3* and *MALAT1* in neurons. *MEG3* displayed one of the most distinct cell type-dependent splicing patterns among ENs, INs, and OPCs, as well as among different AD pathological conditions. Previous studies have reported the potential therapeutic effects of *MEG3* in AD, as overexpression of *MEG3* was shown to improve cognitive disorders, reduce nerve damage, and inhibit astrocyte activation in the hippocampal tissues by inhibiting the PI3K/Akt signaling pathway in rats (Yi et al., 2019). However, another study using human neurons transplanted into mouse brains exposed to Aβ found specific NFT pathology and cell death in human neurons, while mouse neurons showed only mild pathology (Balusu et al., 2022). That study further demonstrated that the induction of *MEG3* in *ex vivo* conditions led to cell necroptosis, unlike in mice, which showed no effect (Balusu et al., 2022). Also, unannotated events of *SYT1* in the 5′ region are actually part of *the SYT1* alternative promoter and are not splicing disruption. This is due to some of *the SYT1* variants had not been annotated in the version of the reference genome that we used, GENCODE V39 (Ensemble 105), while they were annotated in the recent version of the reference genome, GENCODE V42 (Ensemble 108). Our study identified an undocumented splicing event, including an unusual junction in position chr17:29,515,203-29,546,866 of the *TAOK1* gene, which is present in all individuals in control, ADM, and ADH groups. Interestingly, the expression of this unknown transcript is increased in ADH ENs compared to the control and ADM ENs. The region lacked pseudogenes, nonsense RNA, and DNase signals, and the deleted region corresponded to the regulatory region of TAOK1 through ATM-mediated phosphorylation (Raman et al., 2007). This unknown transcript was increased in neurons with NFT in all cortical layers, but the increase was not statistically significant. TAOK1 plays a crucial role in regulating microtubule dynamics and organization, and it can induce apoptotic changes through the activation of JNK, MAPK, and caspases. Furthermore, TAOK1 regulates MAPKs and stimulates the JNK and p38 MAPK signaling pathways. Our findings also show that *TAOK1* expression is increased in neurons with NFT, consistent with its role in regulating tau phosphorylation. Two phosphorylated tau residues (T123 and T427) are identified in the AD brain that appears to be specifically targeted by TAOK1. Notably, a TAOK1 inhibitor reduced tau phosphorylation in cortical neurons without affecting synapse markers and neuronal health (Giacomini et al., 2018). These results suggest that *TAOK1* may be a potential therapeutic target for tauopathy in AD.

On the other hand, it is important to note that not all ASEs of certain genes can be detected in single-cell data due to poly A tail capture. Although almost all transcripts significantly altered in AD have a stop codon in the ORF or 3’ UTR, except for some *CLU* and *TAOK1* transcripts, which are likely to be eliminated by non-stop decay mechanisms, it is still unclear whether these transcripts decayed by other mRNA surveillance mechanisms such as peptide dependent translation arrest, 18S nonfunctional rRNA decay, and mRNAs containing a strong secondary structure within the ORF (Wolin and Maquat, 2019). it is not completely clear which sequence length increase or decrease can affect RNA turnover (Andrzejewska et al., 2020). Furthermore, the question remains whether these ASEs contribute to the development of AD pathology or are a result of it or whether a positive feedback loop is involved. Lastly, it is unclear whether these altered transcripts are translated into functional proteins and, if so, what impact these changes have on protein function in some ASEs. Further investigation is required to address these questions.

In the future, the VASA-Seq technology could be a valuable tool for identifying all relevant ASEs in AD postmortem brain tissues due to its ability to provide full-length reads in over 10,000 cells (Salmen et al., 2022). However, to validate and further investigate the spread of these ASEs as the first steps, more precise methods such as *in situ* hybridization and immunohistochemistry should be used. There is also currently less data available in the databases for CELF2 and DDX5 than for other RBPs. Crosslinking and immunoprecipitation methods could also be employed to explore the interaction between splicing factors and ASEs (Sternburg and Karginov, 2020). The recently developed single-cell long-read method can provide more details of the final RNA sequence and may predict more translational regulation by the asset of the machine learning approach than the current studies (Hardwick et al., 2022; Joglekar et al., 2023). However, single-cell long read transcriptomics has been limited in capturing a wide range of isoform diversity due to the sequencing depth constraints inherent in its protocols. As a result, datasets typically exhibit low redundancy levels between cells of the same cell type (Arzalluz-Luque et al., 2022). Also, polysome profiling provides information about the translational control consequences of ASEs (Reixachs-Solé and Eyras, 2022). Also, after researching these cases, it is possible to integrate RNA sequencing data with other omics data, such as proteomics and epigenomics, and identify potential therapeutic targets. These methods may provide a more complete understanding of the relationship between ASEs and AD pathology, which could lead to the development of novel therapeutic strategies. In summary, our study sheds light on the importance of splicing in the AD pathology that might be considered AD as a spliceopathy during the disease progression. By applying a cell-level approach, we have identified several novel ASEs in the PFC cells of AD. Further research on splicing in AD may lead to the development of novel diagnostic and therapeutic strategies for neurodegenerative diseases.

## Data availability statement

Publicly available datasets, including GSE125050, GSE157827, and GSE129308, available at https://www.ncbi.nlm.nih.gov/, were used in this study. Processed post SICILIAN data in this study, including all true junctions used for the SpliZ pipeline, are openly available in the compressed tsv file for NGE at https://figshare.com/articles/dataset/NGE_prespliz/23522391 and in the compressed tsv file for NFT at https://figshare.com/articles/dataset/NFT_prespilz/23530641. The code used in this study is available through the following repository: https://github.com/MEFarhadieh/SCADSplice.

## Ethics statement

The Brain and Body Donation Program has been supported by the National Institute of Neurological Disorders and Stroke (U24 NS072026, National Brain and Tissue Resource for Parkinson’s Disease and Related Disorders), the National Institute on Aging (P30 AG19610, Arizona Alzheimer’s Disease Core Center), the Arizona Department of Health Services (contract 211002, Arizona Alzheimer’s Research Center), the Arizona Biomedical Research Commission (contracts 4001, 0011, 05-901, and 1001 to the Arizona Parkinson’s Disease Consortium), and the Michael J. Fox Foundation for Parkinson’s Research for GSE125050 dataset. The SWDBB for providing brain tissues for this study. The SWDBB is part of the Brains for Dementia Research program, which is jointly funded by Alzheimer’s Research United Kingdom and the Alzheimer’s Society and is supported by Bristol Research into Alzheimer’s and Care of the Elderly and the Medical Research Council for GSE157827 dataset. Human tissue was obtained from UCLA-Easton Center, NIH Neurobiobank (Sepulveda repository, Los Angeles, CA, and Mount Sinai, New York, NY), and the Stanford Alzheimer Disease Research Center (NIH/NIA P30 AG066515) for GSE129308 dataset. The studies were conducted in accordance with the local legislation and institutional requirements. Written informed consent for participation was not required from the participants or the participants’ legal guardians/next of kin in accordance with the national legislation and institutional requirements.

## Author contributions

M-EF and KG conceptualized and designed study. M-EF collected and analyzed data and wrote manuscript. KG supervised study and reviewed manuscript. All authors contributed to the article and approved the submitted version.

## Conflict of interest

The authors declare that the research was conducted in the absence of any commercial or financial relationships that could be construed as a potential conflict of interest.

## Publisher’s note

All claims expressed in this article are solely those of the authors and do not necessarily represent those of their affiliated organizations, or those of the publisher, the editors and the reviewers. Any product that may be evaluated in this article, or claim that may be made by its manufacturer, is not guaranteed or endorsed by the publisher.

## Footnotes

^1^ https://ftp.ebi.ac.uk/pub/databases/gencode/Gencode_human/release_39/gencode.v39.annotation.gff3.gz

^2^ https://ftp.ebi.ac.uk/pub/databases/gencode/Gencode_human/release_39/gencode.v39.annotation.gtf.gz

^3^ https://genome.ucsc.edu/cgi-bin/hgTables

^4^ https://ftp.ebi.ac.uk/pub/databases/gencode/Gencode_human/release_39/GRCh38.p13.genome.fa.gz

^5^ https://ftp.ebi.ac.uk/pub/databases/gencode/Gencode_human/release_39/gencode.v39.transcripts.fa.gz

^6^ https://hgdownload.soe.ucsc.edu/goldenPath/hg38/database/ucscGenePfam.txt.gz

## References

[ref1] Alves S. S. Silva-Junior R. M. P. Servilha-Menezes G. Homolak J. Šalković-Petrišić M. Garcia-Cairasco N. (2021). Insulin resistance as a common link between current Alzheimer’s disease hypotheses. J. Alzheimers Dis. 82, 71–105. doi: 10.3233/JAD-210234, PMID: 34024838

[ref2] Amezquita R. A. Lun A. T. L. Becht E. Carey V. J. Carpp L. N. Geistlinger L.. (2020). Orchestrating single-cell analysis with Bioconductor. Nat. Methods 17, 137–145. doi: 10.1038/s41592-019-0654-x, PMID: 31792435 PMC7358058

[ref3] Andrzejewska A. Zawadzka M. Pachulska-Wieczorek K. (2020). On the way to understanding the interplay between the RNA structure and functions in cells: a genome-wide perspective. Int. J. Mol. Sci. 21:6770. doi: 10.3390/ijms21186770, PMID: 32942713 PMC7554983

[ref4] Arizaca Maquera K. A. Welden J. R. Margvelani G. Miranda Sardón S. C. Hart S. Robil N.. (2023). Alzheimer’s disease pathogenetic progression is associated with changes in regulated retained introns and editing of circular RNAs. Front. Mol. Neurosci. 16:1141079. doi: 10.3389/fnmol.2023.1141079, PMID: 37266374 PMC10231643

[ref5] Arzalluz-Luque A. Salguero P. Tarazona S. Conesa A. (2022). ACORDE unravels functionally interpretable networks of isoform co-usage from single cell data. Nat. Commun. 13:1828. doi: 10.1038/s41467-022-29497-w, PMID: 35383181 PMC8983708

[ref6] Bai B. Hales C. M. Chen P.-C. Gozal Y. Dammer E. B. Fritz J. J.. (2013). U1 small nuclear ribonucleoprotein complex and RNA splicing alterations in Alzheimer’s disease. Proc. Natl. Acad. Sci. 110, 16562–16567. doi: 10.1073/pnas.1310249110, PMID: 24023061 PMC3799305

[ref7] Balusu S. Horré K. Thrupp N. Snellinx A. Serneels L. Chrysidou I.. (2022). Long noncoding RNA MEG3 activates neuronal necroptosis in Alzheimer’s disease. BioRxiv 14, 2002–2022. doi: 10.1101/2022.02.18.480849

[ref8] Bellenguez C. Küçükali F. Jansen I. E. Kleineidam L. Moreno-Grau S. Amin N.. (2022). New insights into the genetic etiology of Alzheimer’s disease and related dementias. Nat. Genet. 54, 412–436. doi: 10.1038/s41588-022-01024-z, PMID: 35379992 PMC9005347

[ref9] Bhadra M. Howell P. Dutta S. Heintz C. Mair W. B. (2020). Alternative splicing in aging and longevity. Hum. Genet. 139, 357–369. doi: 10.1007/s00439-019-02094-6, PMID: 31834493 PMC8176884

[ref10] Biamonti G. Amato A. Belloni E. Di Matteo A. Infantino L. Pradella D.. (2021). Alternative splicing in Alzheimer’s disease. Aging Clin. Exp. Res. 33, 747–758. doi: 10.1007/s40520-019-01360-x 31583531

[ref11] Bishof I. Dammer E. B. Duong D. M. Kundinger S. R. Gearing M. Lah J. J.. (2018). RNA-binding proteins with basic-acidic dipeptide (BAD) domains self-assemble and aggregate in Alzheimer’s disease. J. Biol. Chem. 293, 11047–11066. doi: 10.1074/jbc.RA118.001747, PMID: 29802200 PMC6052236

[ref12] Bohush A. Bieganowski P. Filipek A. (2019). Hsp90 and its co-chaperones in neurodegenerative diseases. Int. J. Mol. Sci. 20:4976. doi: 10.3390/ijms20204976, PMID: 31600883 PMC6834326

[ref13] Breijyeh Z. Karaman R. (2020). Comprehensive review on Alzheimer’s disease: causes and treatment. Molecules 25:5789. doi: 10.3390/molecules25245789, PMID: 33302541 PMC7764106

[ref14] Chabot B. Shkreta L. (2016). Defective control of pre–messenger RNA splicing in human disease. J. Cell Biol. 212, 13–27. doi: 10.1083/jcb.201510032, PMID: 26728853 PMC4700483

[ref15] Dai L. Chen K. Youngren B. Kulina J. Yang A. Guo Z.. (2016). Cytoplasmic Drosha activity generated by alternative splicing. Nucleic Acids Res. 44, 10454–10466. doi: 10.1093/nar/gkw668, PMID: 27471035 PMC5137420

[ref16] Dehghannasiri R. Olivieri J. E. Damljanovic A. Salzman J. (2021). Specific splice junction detection in single cells with SICILIAN. Genome Biol. 22, 1–13. doi: 10.1186/s13059-021-02434-8 34353340 PMC8339681

[ref17] Deschenes M. Chabot B. (2017). The emerging role of alternative splicing in senescence and aging. Aging Cell 16, 918–933. doi: 10.1111/acel.12646, PMID: 28703423 PMC5595669

[ref18] Dharshini S. A. P. Taguchi Y. H. Gromiha M. M. (2019). Exploring the selective vulnerability in Alzheimer disease using tissue specific variant analysis. Genomics 111, 936–949. doi: 10.1016/j.ygeno.2018.05.024, PMID: 29879491

[ref19] Dobin A. Davis C. A. Schlesinger F. Drenkow J. Zaleski C. Jha S.. (2013). STAR: ultrafast universal RNA-seq aligner. Bioinformatics 29, 15–21. doi: 10.1093/bioinformatics/bts635, PMID: 23104886 PMC3530905

[ref20] Ewels P. A. Peltzer A. Fillinger S. Patel H. Alneberg J. Wilm A.. (2020). The nf-core framework for community-curated bioinformatics pipelines. Nat. Biotechnol. 38, 276–278. doi: 10.1038/s41587-020-0439-x, PMID: 32055031

[ref21] Foster E. M. Dangla-Valls A. Lovestone S. Ribe E. M. Buckley N. J. (2019). Clusterin in Alzheimer’s disease: mechanisms, genetics, and lessons from other pathologies. Front. Neurosci. 13:164. doi: 10.3389/fnins.2019.00164, PMID: 30872998 PMC6403191

[ref22] Gabut M. Samavarchi-Tehrani P. Wang X. Slobodeniuc V. O’Hanlon D. Sung H.-K.. (2011). An alternative splicing switch regulates embryonic stem cell pluripotency and reprogramming. Cells 147, 132–146. doi: 10.1016/j.cell.2011.08.023 21924763

[ref23] Giacomini C. Koo C.-Y. Yankova N. Tavares I. A. Wray S. Noble W.. (2018). A new TAO kinase inhibitor reduces tau phosphorylation at sites associated with neurodegeneration in human tauopathies. Acta Neuropathol. Commun. 6, 1–16. doi: 10.1186/s40478-018-0539-8 29730992 PMC5937037

[ref24] Gupta A. Shamsi F. Altemose N. Dorlhiac G. F. Cypess A. M. White A. P.. (2022). Characterization of transcript enrichment and detection bias in single-nucleus RNA-seq for mapping of distinct human adipocyte lineages. Genome Res. 32, 242–257. doi: 10.1101/gr.275509.121, PMID: 35042723 PMC8805720

[ref25] Gustavsson A. Norton N. Fast T. Frölich L. Georges J. Holzapfel D.. (2023). Global estimates on the number of persons across the Alzheimer’s disease continuum. Alzheimers Dement. 19, 658–670. doi: 10.1002/alz.12694, PMID: 35652476

[ref26] Hales C. M. Dammer E. B. Diner I. Yi H. Seyfried N. T. Gearing M.. (2014). Aggregates of Small nuclear ribonucleic acids (snRNAs) in a lzheimer’s disease. Brain Pathol. 24, 344–351. doi: 10.1111/bpa.12133, PMID: 24571648 PMC4096308

[ref27] Hao Y. Hao S. Andersen-Nissen E. Mauck W. M. III Zheng S. Butler A.. (2021). Integrated analysis of multimodal single-cell data. Cells 184, 3573–3587.e29. doi: 10.1016/j.cell.2021.04.048, PMID: 34062119 PMC8238499

[ref28] Hardwick S. A. Hu W. Joglekar A. Fan L. Collier P. G. Foord C.. (2022). Single-nuclei isoform RNA sequencing unlocks barcoded exon connectivity in frozen brain tissue. Nat. Biotechnol. 40, 1082–1092. doi: 10.1038/s41587-022-01231-3, PMID: 35256815 PMC9287170

[ref29] He L. Loika Y. Kulminski A. M. (2022). Allele-specific analysis reveals exon-and cell-type-specific regulatory effects of Alzheimer’s disease-associated genetic variants. Transl. Psychiatry 12:163. doi: 10.1038/s41398-022-01913-1, PMID: 35436980 PMC9016079

[ref30] Herrup K. (2021). How Not To Study a Disease: The Story of Alzheimer’s. Cambridge, MA: MIT Press.

[ref31] Hodge R. D. Bakken T. E. Miller J. A. Smith K. A. Barkan E. R. Graybuck L. T.. (2019). Conserved cell types with divergent features in human versus mouse cortex. Nature 573, 61–68. doi: 10.1038/s41586-019-1506-7, PMID: 31435019 PMC6919571

[ref32] Huynh T.-P. V. Davis A. A. Ulrich J. D. Holtzman D. M. (2017). Apolipoprotein E and Alzheimer’s disease: the influence of apolipoprotein E on amyloid-β and other amyloidogenic proteins: thematic review series: ApoE and lipid homeostasis in Alzheimer’s disease. J. Lipid Res. 58, 824–836. doi: 10.1194/jlr.R075481, PMID: 28246336 PMC5408619

[ref33] Joglekar A. Foord C. Jarroux J. Pollard S. Tilgner H. U. (2023). From words to complete phrases: insight into single-cell isoforms using short and long reads. Transcription, 1–13. doi: 10.1080/21541264.2023.2213514 [Epub ahead of print]., PMID: 37314295 PMC10807471

[ref34] Kannaian B. Sharma B. Phillips M. Chowdhury A. Manimekalai M. S. S. Adav S. S.. (2019). Abundant neuroprotective chaperone Lipocalin-type prostaglandin D synthase (L-PGDS) disassembles the amyloid-β fibrils. Sci. Rep. 9:12579. doi: 10.1038/s41598-019-48819-5, PMID: 31467325 PMC6715741

[ref35] Kar A. Fushimi K. Zhou X. Ray P. Shi C. Chen X.. (2011). RNA helicase p68 (DDX5) regulates tau exon 10 splicing by modulating a stem-loop structure at the 5′ splice site. Mol. Cell. Biol. 31, 1812–1821. doi: 10.1128/MCB.01149-10, PMID: 21343338 PMC3133221

[ref36] Khozoie C. Fancy N. Marjaneh M. M. Murphy A. E. Matthews P. M. Skene N. (2021). ScFlow: a scalable and reproducible analysis pipeline for single-cell RNA sequencing data. bioRxiv, 2008–2021. doi: 10.1101/2021.08.16.456499 [Epub ahead of print].

[ref37] Kim N. Kang H. Jo A. Yoo S.-A. Lee H.-O. (2023). Perspectives on single-nucleus RNA sequencing in different cell types and tissues. J. Pathol. Transl. Med. 57, 52–59. doi: 10.4132/jptm.2022.12.19, PMID: 36623812 PMC9846005

[ref38] Knopman D. S. Amieva H. Petersen R. C. Chételat G. Holtzman D. M. Hyman B. T.. (2021). Alzheimer disease. Nat. Rev. Dis. Prim. 7:33. doi: 10.1038/s41572-021-00269-y, PMID: 33986301 PMC8574196

[ref39] Koh J.-Y. Lee S.-J. (2020). Metallothionein-3 as a multifunctional player in the control of cellular processes and diseases. Mol. Brain 13, 1–12. doi: 10.1186/s13041-020-00654-w 32843100 PMC7448430

[ref40] Lau S.-F. Cao H. Fu A. K. Y. Ip N. Y. (2020). Single-nucleus transcriptome analysis reveals dysregulation of angiogenic endothelial cells and neuroprotective glia in Alzheimer’s disease. Proc. Natl. Acad. Sci. 117, 25800–25809. doi: 10.1073/pnas.2008762117, PMID: 32989152 PMC7568283

[ref41] Lawrence M. Gentleman R. Carey V. (2009). Rtracklayer: an R package for interfacing with genome browsers. Bioinformatics 25, 1841–1842. doi: 10.1093/bioinformatics/btp328, PMID: 19468054 PMC2705236

[ref42] Li M. Geng R. Li C. Meng F. Zhao H. Liu J.. (2021). Dysregulated gene-associated biomarkers for Alzheimer’s disease and aging. Transl. Neurosci. 12, 83–95. doi: 10.1515/tnsci-2021-0009, PMID: 33623715 PMC7885957

[ref43] Link S. Grund S. E. Diederichs S. (2016). Alternative splicing affects the subcellular localization of Drosha. Nucleic Acids Res. 44, 5330–5343. doi: 10.1093/nar/gkw400, PMID: 27185895 PMC4914122

[ref44] Liu J. Guo Z.-N. Yan X.-L. Yang Y. Huang S. (2021). Brain pathogenesis and potential therapeutic strategies in myotonic dystrophy type 1. Front. Aging Neurosci. 13:755392. doi: 10.3389/fnagi.2021.755392, PMID: 34867280 PMC8634727

[ref45] Love M. I. Huber W. Anders S. (2014). Moderated estimation of fold change and dispersion for RNA-seq data with DESeq2. Genome Biol. 15, 1–21. doi: 10.1186/s13059-014-0550-8 PMC4302049 25516281

[ref46] Lu Y. Yue D. Xie J. Cheng L. Wang X. (2022). Ontology specific alternative splicing changes in Alzheimer’s disease. Front. Genet. 13:49. doi: 10.3389/fgene.2022.926049, PMID: 35774499 PMC9237535

[ref47] Maciel P. Cruz V. T. Constante M. Iniesta I. Costa M. D. C. Gallati S.. (2005). Neuroferritinopathy: missense mutation in FTL causing early-onset bilateral pallidal involvement. Neurology 65, 603–605. doi: 10.1212/01.wnl.0000178224.81169.c2, PMID: 16116125 PMC2886026

[ref48] Marques-Coelho D. Iohan L. D. C. C. Melo de Farias A. R. Flaig A. Lambert J.-C. Costa M. R. (2021). Differential transcript usage unravels gene expression alterations in Alzheimer’s disease human brains. NPJ Aging Mech. Dis. 7:2. doi: 10.1038/s41514-020-00052-5, PMID: 33398016 PMC7782705

[ref49] Matera A. G. Wang Z. (2014). A day in the life of the spliceosome. Nat. Rev. Mol. Cell Biol. 15, 108–121. doi: 10.1038/nrm3742, PMID: 24452469 PMC4060434

[ref50] Maziuk B. F. Apicco D. J. Cruz A. L. Jiang L. Ash P. E. A. da Rocha E. L.. (2018). RNA binding proteins co-localize with small tau inclusions in tauopathy. Acta Neuropathol. Commun. 6, 1–14. doi: 10.1186/s40478-018-0574-5 30068389 PMC6069705

[ref51] Meyer E. Chaung K. Dehghannasiri R. Salzman J. (2022). ReadZS detects cell type-specific and developmentally regulated RNA processing programs in single-cell RNA-seq. Genome Biol. 23, 1–28. doi: 10.1186/s13059-022-02795-8 36284317 PMC9594907

[ref52] Muppirala U. K. Honavar V. G. Dobbs D. (2011). Predicting RNA-protein interactions using only sequence information. BMC Bioinformatics 12, 1–11. doi: 10.1186/1471-2105-12-489 22192482 PMC3322362

[ref53] Ochiai Y. Uchida Y. Ohtsuki S. Tachikawa M. Aizawa S. Terasaki T. (2017). The blood-brain barrier fatty acid transport protein 1 (FATP 1/SLC 27A1) supplies docosahexaenoic acid to the brain, and insulin facilitates transport. J. Neurochem. 141, 400–412. doi: 10.1111/jnc.13943, PMID: 28035674

[ref54] Ochiai Y. Uchida Y. Tachikawa M. Couraud P. Terasaki T. (2019). Amyloid beta25-35 impairs docosahexaenoic acid efflux by down-regulating fatty acid transport protein 1 (FATP1/SLC27A1) protein expression in human brain capillary endothelial cells. J. Neurochem. 150, 385–401. doi: 10.1111/jnc.14722, PMID: 31091338

[ref55] Olivieri J. E. Dehghannasiri R. Salzman J. (2022). The SpliZ generalizes ‘percent spliced in’to reveal regulated splicing at single-cell resolution. Nat. Methods 19, 307–310. doi: 10.1038/s41592-022-01400-x, PMID: 35241832 PMC9089759

[ref56] Otero-Garcia M. Mahajani S. U. Wakhloo D. Tang W. Xue Y.-Q. Morabito S.. (2022). Molecular signatures underlying neurofibrillary tangle susceptibility in Alzheimer’s disease. Neuron 110, 2929–2948.e8. doi: 10.1016/j.neuron.2022.06.021, PMID: 35882228 PMC9509477

[ref57] Patrick R. Humphreys D. T. Janbandhu V. Oshlack A. Ho J. W. K. Harvey R. P.. (2020). Sierra: discovery of differential transcript usage from polyA-captured single-cell RNA-seq data. Genome Biol. 21, 1–27. doi: 10.1186/s13059-020-02071-7 PMC7341584 32641141

[ref58] Patro R. Duggal G. Love M. I. Irizarry R. A. Kingsford C. (2017). Salmon provides fast and bias-aware quantification of transcript expression. Nat. Methods 14, 417–419. doi: 10.1038/nmeth.4197, PMID: 28263959 PMC5600148

[ref59] Pavan R. R. Diniz F. El-Dahr S. Tortelote G. G. (2023). Gene length is a pivotal feature to explain disparities in transcript capture between single transcriptome techniques. Front. Bioinforma. 3:1144266. doi: 10.3389/fbinf.2023.1144266, PMID: 37122996 PMC10132733

[ref60] Penney J. Ralvenius W. T. Tsai L.-H. (2020). Modeling Alzheimer’s disease with iPSC-derived brain cells. Mol. Psychiatry 25, 148–167. doi: 10.1038/s41380-019-0468-3, PMID: 31391546 PMC6906186

[ref61] Raj T. Li Y. I. Wong G. Humphrey J. Wang M. Ramdhani S.. (2018). Integrative transcriptome analyses of the aging brain implicate altered splicing in Alzheimer’s disease susceptibility. Nat. Genet. 50, 1584–1592. doi: 10.1038/s41588-018-0238-1, PMID: 30297968 PMC6354244

[ref62] Raman M. Earnest S. Zhang K. Zhao Y. Cobb M. H. (2007). TAO kinases mediate activation of p38 in response to DNA damage. EMBO J. 26, 2005–2014. doi: 10.1038/sj.emboj.7601668, PMID: 17396146 PMC1852793

[ref63] Reixachs-Solé M. Eyras E. (2022). Uncovering the impacts of alternative splicing on the proteome with current omics techniques. Wiley Interdiscip. Rev. RNA 13:e1707. doi: 10.1002/wrna.1707, PMID: 34979593 PMC9542554

[ref64] Salmen F. De Jonghe J. Kaminski T. S. Alemany A. Parada G. E. Verity-Legg J.. (2022). High-throughput total RNA sequencing in single cells using VASA-seq. Nat. Biotechnol. 40, 1780–1793. doi: 10.1038/s41587-022-01361-8 35760914 PMC9750877

[ref65] Sharma A. Sethi G. Tambuwala M. M. Aljabali A. A. A. Chellappan D. K. Dua K.. (2021). Circadian rhythm disruption and Alzheimer’s disease: the dynamics of a vicious cycle. Curr. Neuropharmacol. 19, 248–264. doi: 10.2174/18756190MTA21MjAf3, PMID: 32348224 PMC8033974

[ref66] Shen S. Park J. W. Lu Z. Lin L. Henry M. D. Wu Y. N.. (2014). rMATS: robust and flexible detection of differential alternative splicing from replicate RNA-Seq data. Proc. Natl. Acad. Sci. 111, E5593–E5601. doi: 10.1073/pnas.1419161111 25480548 PMC4280593

[ref67] Sluysmans S. Méan I. Xiao T. Boukhatemi A. Ferreira F. Jond L.. (2021). PLEKHA5, PLEKHA6, and PLEKHA7 bind to PDZD11 to target the Menkes ATPase ATP7A to the cell periphery and regulate copper homeostasis. Mol. Biol. Cell 32:ar34. doi: 10.1091/mbc.E21-07-0355, PMID: 34613798 PMC8693958

[ref68] Smith T. Heger A. Sudbery I. (2017). UMI-tools: modeling sequencing errors in unique molecular identifiers to improve quantification accuracy. Genome Res. 27, 491–499. doi: 10.1101/gr.209601.116, PMID: 28100584 PMC5340976

[ref69] Srinivasan K. Friedman B. A. Etxeberria A. Huntley M. A. van Der Brug M. P. Foreman O.. (2020). Alzheimer’s patient microglia exhibit enhanced aging and unique transcriptional activation. Cell Rep. 31:107843. doi: 10.1016/j.celrep.2020.107843, PMID: 32610143 PMC7422733

[ref70] Sternburg E. L. Karginov F. V. (2020). Global approaches in studying RNA-binding protein interaction networks. Trends Biochem. Sci. 45, 593–603. doi: 10.1016/j.tibs.2020.03.005, PMID: 32531229

[ref71] Szklarczyk D. Kirsch R. Koutrouli M. Nastou K. Mehryary F. Hachilif R.. (2023). The STRING database in 2023: protein–protein association networks and functional enrichment analyses for any sequenced genome of interest. Nucleic Acids Res. 51, D638–D646. doi: 10.1093/nar/gkac1000, PMID: 36370105 PMC9825434

[ref72] Tekath T. Dugas M. (2021). Differential transcript usage analysis of bulk and single-cell RNA-seq data with DTUrtle. Bioinformatics 37, 3781–3787. doi: 10.1093/bioinformatics/btab629, PMID: 34469510 PMC8570804

[ref73] Tran B. K. (2023). Understanding the Role of CELF in Alzheimer’s Disease Using *C. elegans*. Doctoral dissertation, The University of Texas Health Science Center at San Antonio.

[ref74] Tuvshinjargal N. Lee W. Park B. Han K. (2016). PRIdictor: protein–RNA interaction predictor. Biosystems 139, 17–22. doi: 10.1016/j.biosystems.2015.10.004, PMID: 26607710

[ref75] Unno K. Konishi T. Nakagawa A. Narita Y. Takabayashi F. Okamura H.. (2015). Cognitive dysfunction and amyloid β accumulation are ameliorated by the ingestion of green soybean extract in aged mice. J. Funct. Foods 14, 345–353. doi: 10.1016/j.jff.2015.02.011

[ref76] Vanderweyde T. Apicco D. J. Youmans-Kidder K. Ash P. E. A. Cook C. da Rocha E. L.. (2016). Interaction of tau with the RNA-binding protein TIA1 regulates tau pathophysiology and toxicity. Cell Rep. 15, 1455–1466. doi: 10.1016/j.celrep.2016.04.045, PMID: 27160897 PMC5325702

[ref77] Walter W. Sánchez-Cabo F. Ricote M. (2015). GOplot: an R package for visually combining expression data with functional analysis. Bioinformatics 31, 2912–2914. doi: 10.1093/bioinformatics/btv300, PMID: 25964631

[ref78] Welch J. D. Kozareva V. Ferreira A. Vanderburg C. Martin C. Macosko E. Z. (2019). Single-cell multi-omic integration compares and contrasts features of brain cell identity. Cells 177, 1873–1887.e17. doi: 10.1016/j.cell.2019.05.006, PMID: 31178122 PMC6716797

[ref79] Weller A. E. Doyle G. A. Reiner B. C. Crist R. C. Berrettini W. H. (2022). Analysis of differential gene expression and transcript usage in hippocampus of Apoe null mutant mice: implications for Alzheimer’s disease. Neurosci. Res. 176, 85–89. doi: 10.1016/j.neures.2021.10.010, PMID: 34757086 PMC8960320

[ref80] Westoby J. Artemov P. Hemberg M. Ferguson-Smith A. (2020). Obstacles to detecting isoforms using full-length scRNA-seq data. Genome Biol. 21, 1–19. doi: 10.1186/s13059-020-01981-w PMC7087381 32293520

[ref81] Wolin S. L. Maquat L. E. (2019). Cellular RNA surveillance in health and disease. Science 366, 822–827. doi: 10.1126/science.aax2957 31727827 PMC6938259

[ref82] Xu H. Liu X. Li W. Xi Y. Su P. Meng B.. (2021). p38 MAPK-mediated loss of nuclear RNase III enzyme Drosha underlies amyloid beta-induced neuronal stress in Alzheimer’s disease. Aging Cell 20:e13434. doi: 10.1111/acel.13434, PMID: 34528746 PMC8521488

[ref83] Yanaizu M. Washizu C. Nukina N. Satoh J. Kino Y. (2020). CELF2 regulates the species-specific alternative splicing of TREM2. Sci. Rep. 10:17995. doi: 10.1038/s41598-020-75057-x, PMID: 33093587 PMC7582162

[ref84] Yang M. Ke Y. Kim P. Zhou X. (2021). ExonSkipAD provides the functional genomic landscape of exon skipping events in Alzheimer’s disease. Brief. Bioinform. 22:bbaa438. doi: 10.1093/bib/bbaa438, PMID: 33497435 PMC8425305

[ref85] Yi J. Chen B. Yao X. Lei Y. Ou F. Huang F. (2019). Upregulation of the lncRNA MEG3 improves cognitive impairment, alleviates neuronal damage, and inhibits activation of astrocytes in hippocampus tissues in Alzheimer’s disease through inactivating the PI3K/Akt signaling pathway. J. Cell. Biochem. 120, 18053–18065. doi: 10.1002/jcb.29108, PMID: 31190362

[ref86] Zaghlool A. Niazi A. Björklund Å. K. Westholm J. O. Ameur A. Feuk L. (2021). Characterization of the nuclear and cytosolic transcriptomes in human brain tissue reveals new insights into the subcellular distribution of RNA transcripts. Sci. Rep. 11:4076. doi: 10.1038/s41598-021-83541-1, PMID: 33603054 PMC7893067

[ref87] Zhang X. Chen M. H. Wu X. Kodani A. Fan J. Doan R.. (2016). Cell-type-specific alternative splicing governs cell fate in the developing cerebral cortex. Cells 166, 1147–1162.e15. doi: 10.1016/j.cell.2016.07.025, PMID: 27565344 PMC5248659

[ref88] Zuehlke A. D. Beebe K. Neckers L. Prince T. (2015). Regulation and function of the human HSP90AA1 gene. Gene 570, 8–16. doi: 10.1016/j.gene.2015.06.018, PMID: 26071189 PMC4519370

