# Supplementary material for: Analyzing alternative splicing in Alzheimer’s disease postmortem brain: a cell-level perspective
Source: Front Mol Neurosci. 2023 Sep 20;16:1237874. doi: 10.3389/fnmol.2023.1237874 (PMC10548223; doi:10.3389/fnmol.2023.1237874)
Supplement: Supplementary file 2 [file Data_Sheet_2.DOCX]

Supplementary Material

Analyzing Alternative Splicing in Alzheimer's Disease Postmortem Brain: A Cell-Level Perspective

Mohammad-Erfan Farhadieh ^1^, Kamran Ghaedi^1*^

*** Correspondence:** Kamran Ghaedi Author: Kamranghaedi@sci.ui.ac.ir

# Supplementary Figures and Tables

## Supplementary Figures


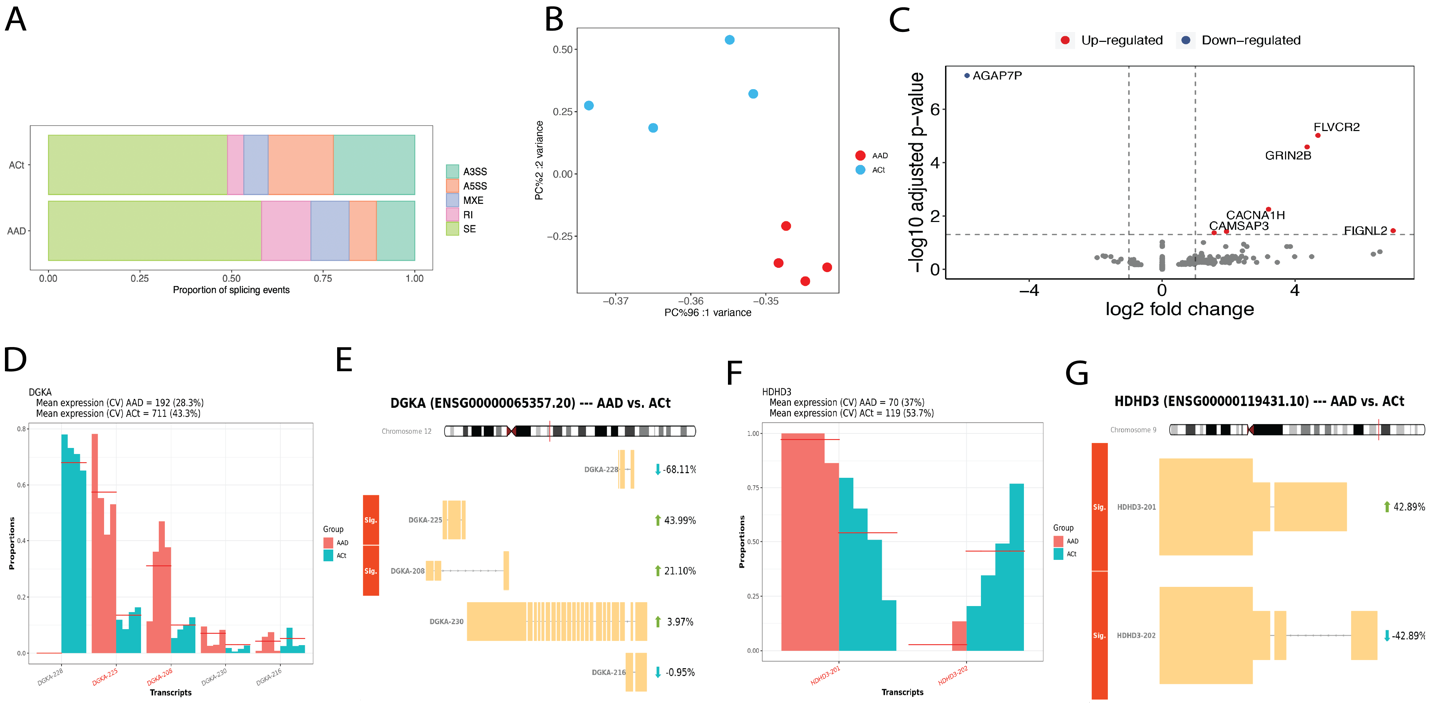


**Supplementary Figure S1** ASEs and DTU of astrocyte (**A**) proportion of ASE in Alzheimer’s disease (AAD) and control (ACt) group. (**B**) The PCA plot of the PSI values. AAD and ACt groups were clearly classified. (**C**) Volcano plot of DGE. The Most significant genes are labeled. (**D-G**) The most significant genes by gene q value and minimal transcript q value are presented by proportion bar plot and genomic position, respectively. Significant changes in transcript expression are indicated by red color and sign.


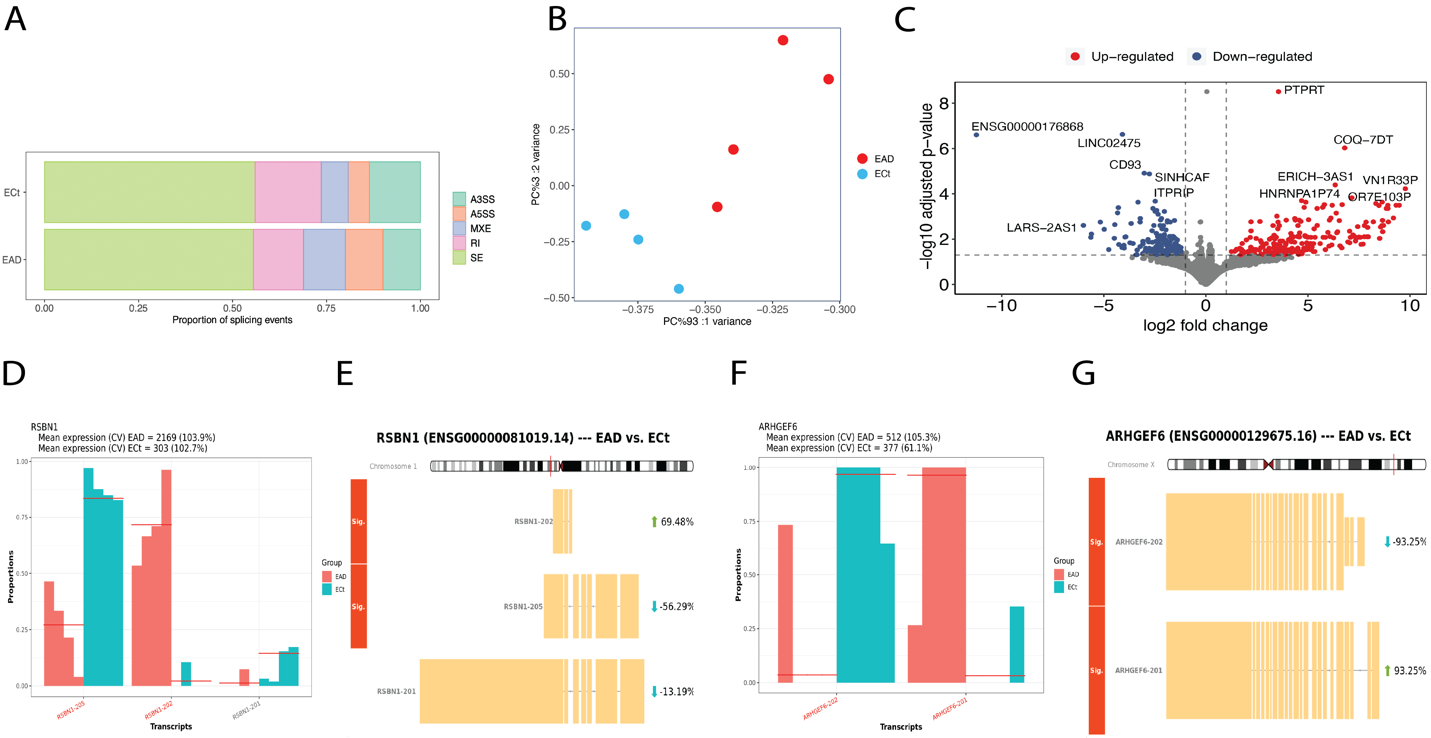


**Supplementary Figure S2** ASEs and DTU of endothelial cells (**A**) proportion of ASE in Alzheimer’s disease (EAD) and control (ECt) group. (**B**) The PCA plot of the PSI values. EAD and ECt groups were clearly classified. (**C**) Volcano plot of DGE. The Most significant genes are labeled. (**D-G**) The most significant genes by gene q value and minimal transcript q value are presented by proportion bar plot and genomic position, respectively. Significant changes in transcript expression are indicated by red color and sign.


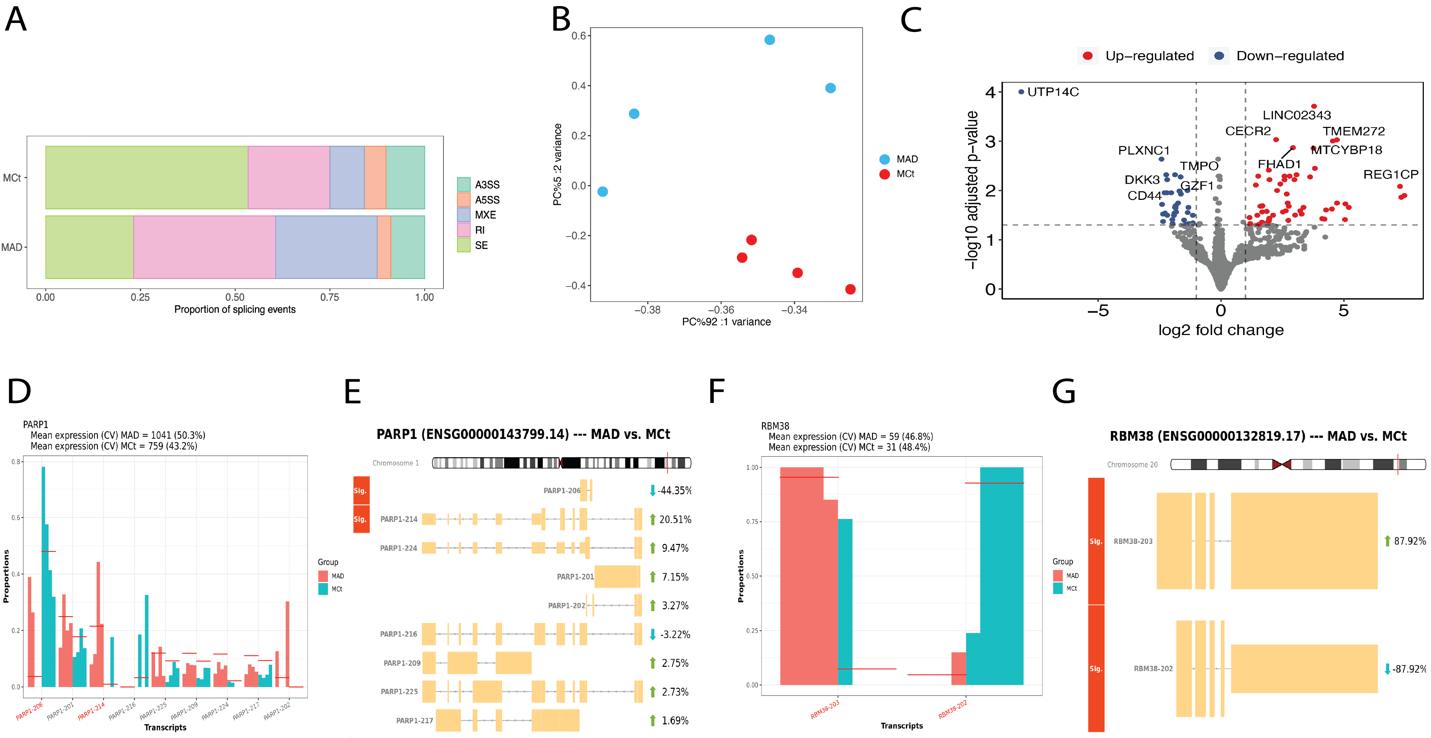


**Supplementary Figure S3** ASEs and DTU of microglia (**A**) proportion of ASE in Alzheimer’s disease (MAD) and control (MCt) group. (**B**) The PCA plot of the PSI values. MAD and MCt groups were clearly classified. (**C**) Volcano plot of DGE. The Most significant genes are labeled. (**D-G**) The most significant genes by gene q value and minimal transcript q value are presented by proportion bar plot and genomic position, respectively. Significant changes in transcript expression are indicated by red color and sign.


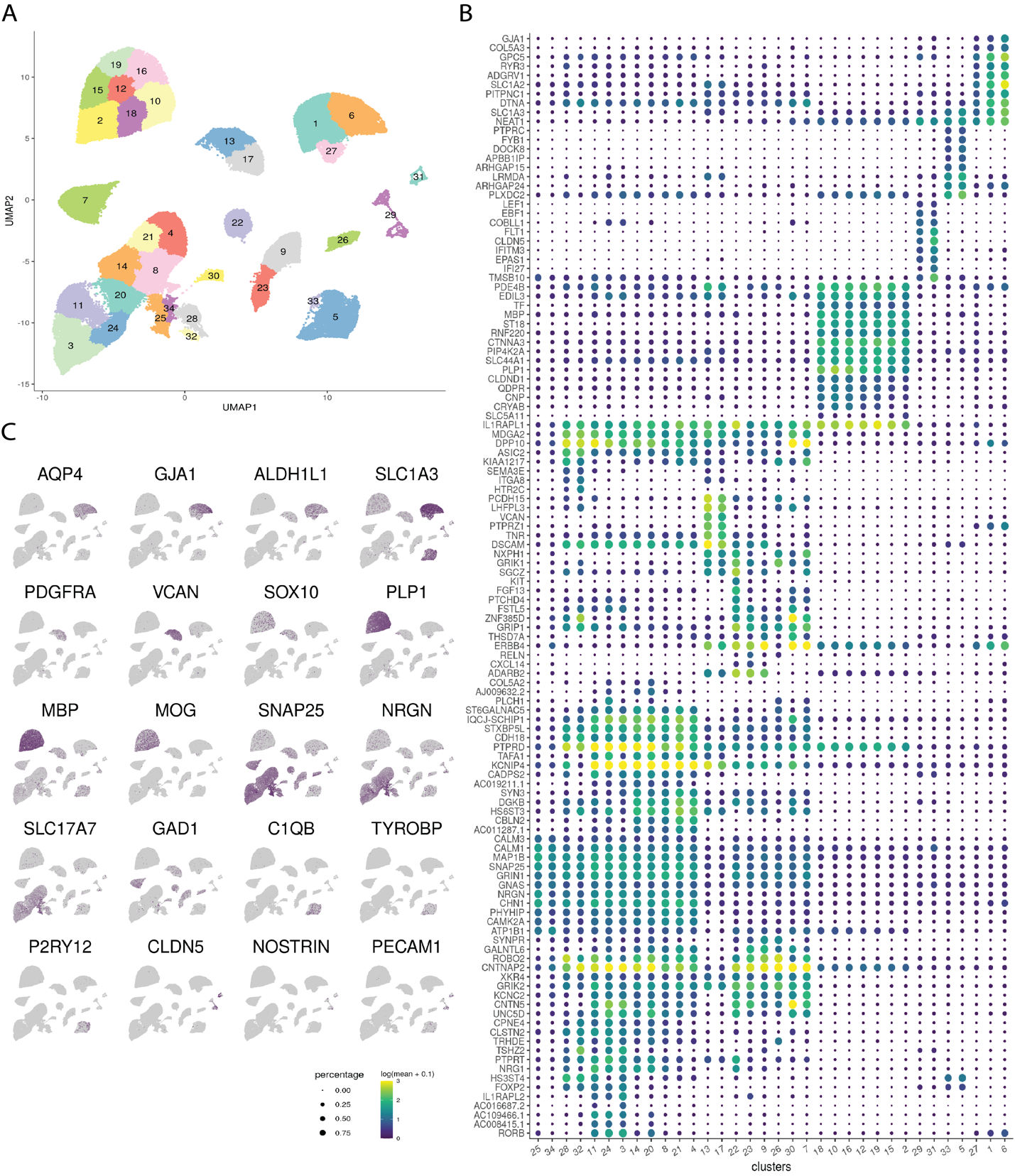


**Supplementary Figure S4** Clustering NGE data. (**A**) UMAP of clusters. (**B**) Top cluster marker genes plot for cells in PFC. (**C**) UMAP feature plots of canonical cell marker genes for well-known cell types.


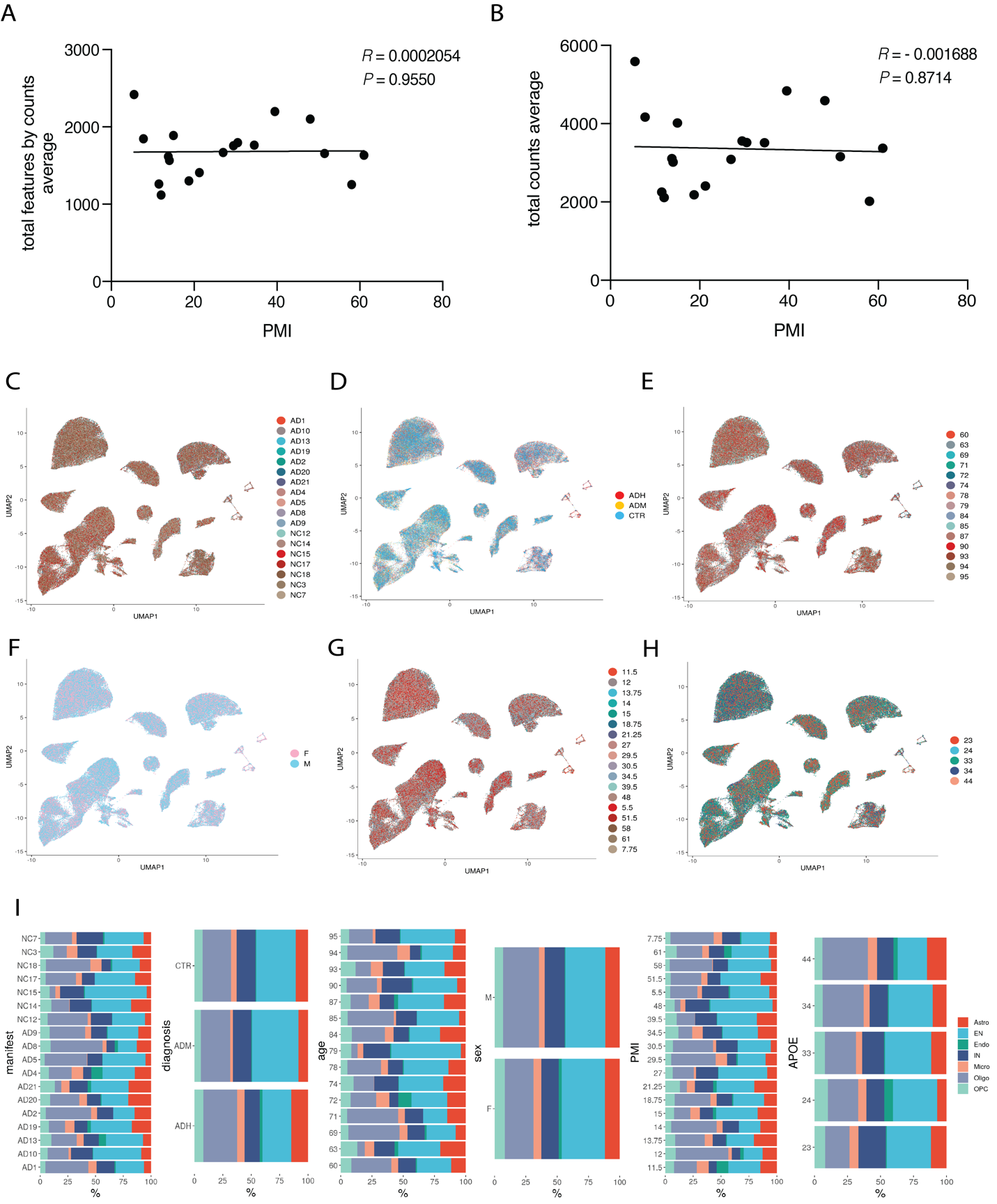


**Supplementary Figure S5** Assessment of NGE donor’s metavariables. (**A**) Correlation between the average number of genes and (**B**) transcripts per cell and post-mortem interval (PMI) in all samples. (**C-H**) UMAP plots show that no cluster was explicitly driven by (**C**) donors, (**D**) diagnosis, (**E**) age, (**F**) gender, (**G**) PMI, and (**H**) APOE genotypes. (**I**) Proportions each cell type per variable.


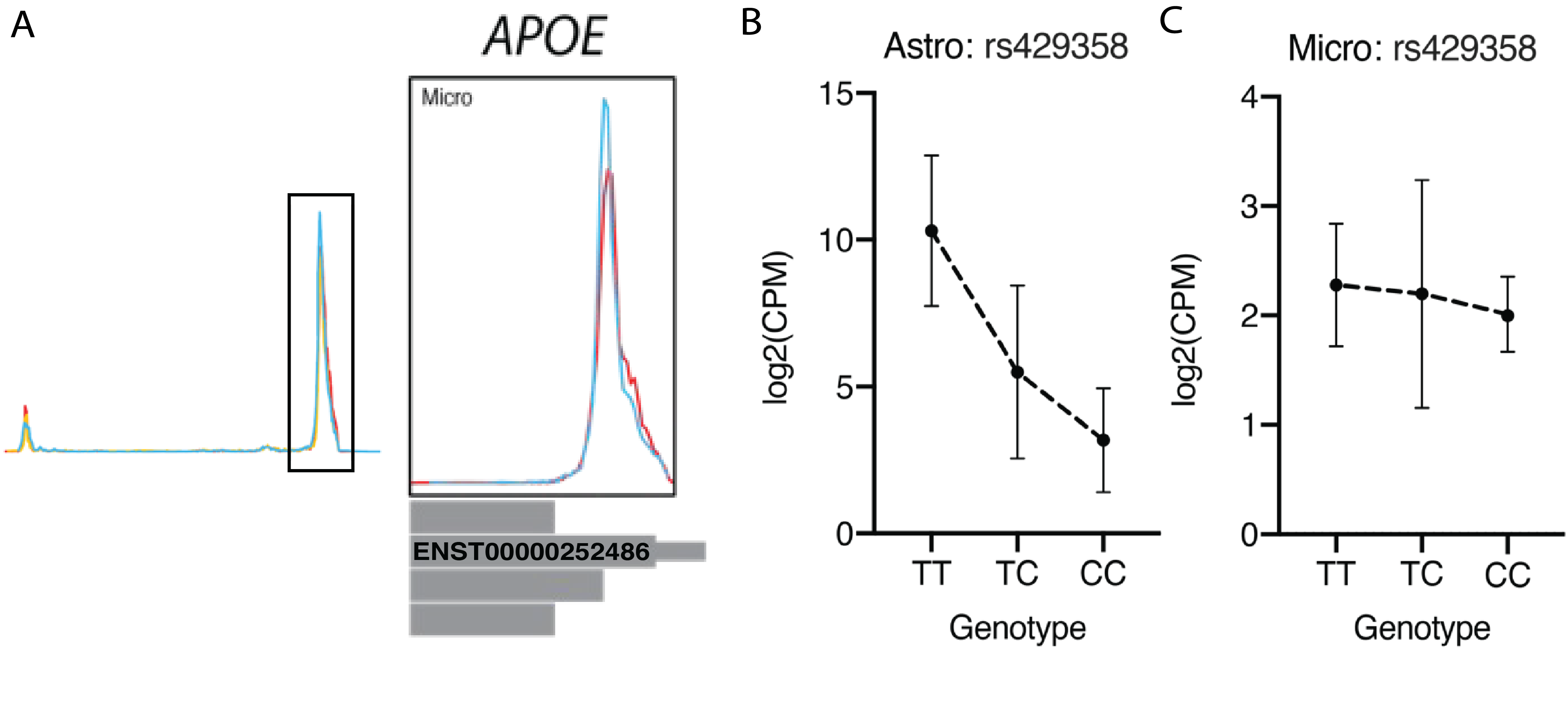


**Supplementary Figure S6** There is no significant correlation between rs429358-C and the expression level of ENST00000252486. (**A**) Peak calling of *APOE* reads in microglia. ADM reads (yellow) is removed in the enlarged frame, and only ADH reads (red) and control reads (blue) is shown in the enlarged frame. (**B, C**) expression level in count per million (CPM) against rs429358 genotypes in (**B**) astrocytes (*P* = 0.1594) and (**C**) microglia (*P* = 0.8624).


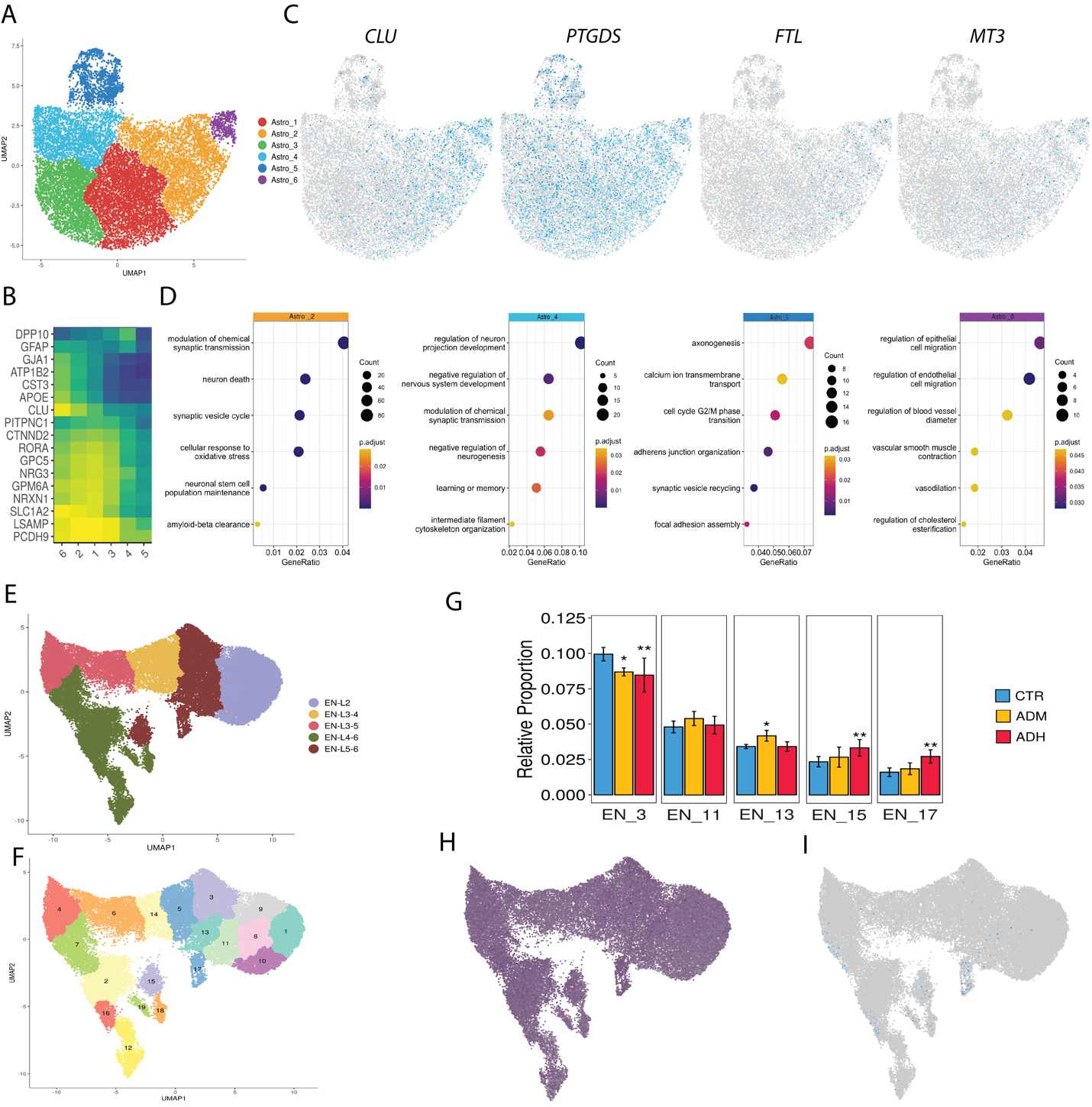


**Supplementary Figure S7** ASEs of NGE cell-subtype level. (**A**) UMAP diagram of astrocyte clusters. (**B**) The heat map of marker genes. Expression scale in terms of logarithms. (**C**) UMAP diagrams of scZ scores of *FTL*, *MT1E*, *MT1G*, and *MT3* in astrocyte population. The intensity of the blue color indicates the deviation from the median of the control state. (**D**) impacted biological pathways in astrocyte subtypes. The number of genes observed in each pathway is directly related to the diameter of the circles. The significance of each path is indicated by a colored bar on the right side of each graph. (**E**) The UMAP diagram of the types of low EN clusters according to the location in the prefrontal cortex layers. (**F**) The UMAP diagram of EN clusters. (**G**) The bar plots of the relative proportion of EN-L5-6 clusters by the group. (**H**) The UMAP of the expression level of *SYT1* in EN subtypes. (I) The UMAP of scZ scores of *SYT1* in EN subtypes. The intensity of the blue color indicates the deviation from the median of the control state.


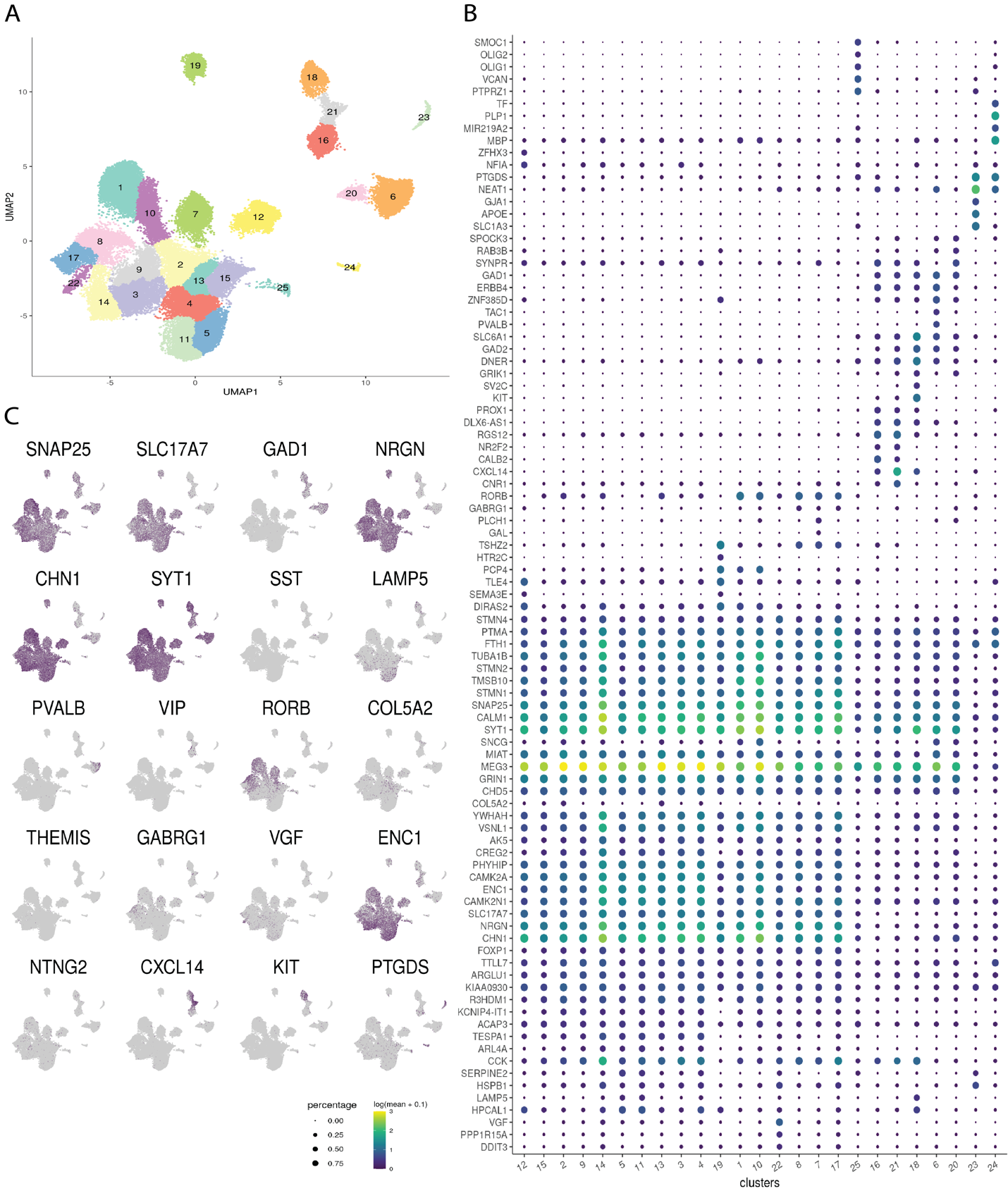


**Supplementary Figure S8** Clustering NFT data. (**A**) UMAP of clusters. (**B**) Top cluster marker genes plot for cells in PFC. (**C**) UMAP feature plots of canonical cell marker genes for well-known cell types.


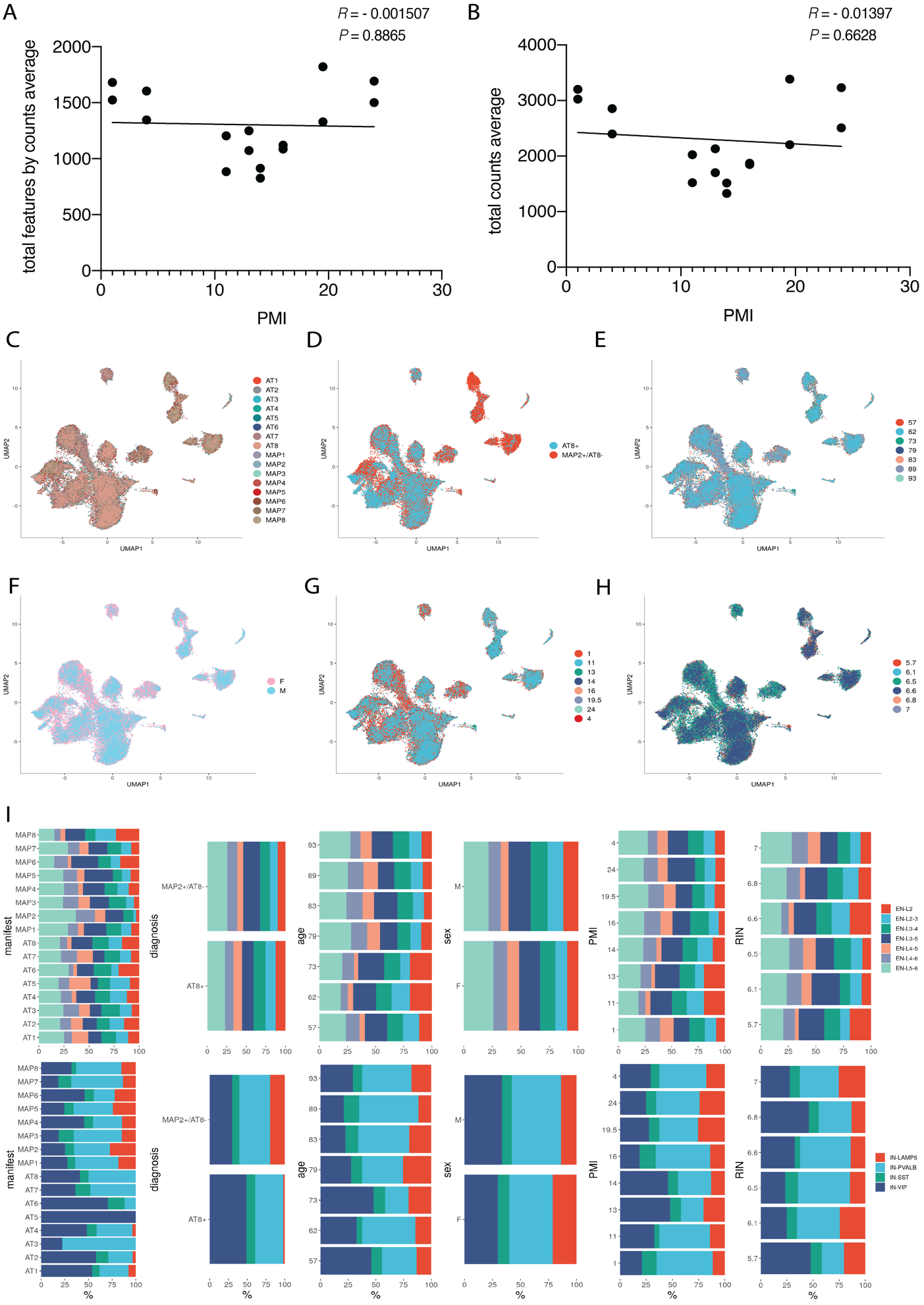


**Supplementary Figure S9** Assessment of NFT’s donor metavariables. (**A**) Correlation between the average number of genes and (**B**) transcripts per cell and post-mortem interval (PMI) in all samples. (**C-H**) UMAP plots show that no cluster was explicitly driven by (**C**) donors, (**D**) diagnosis, (**E**) age, (**F**) gender, (**G**) PMI, and (**H**) APOE genotypes. (**I**) Proportions each cell type per variable.


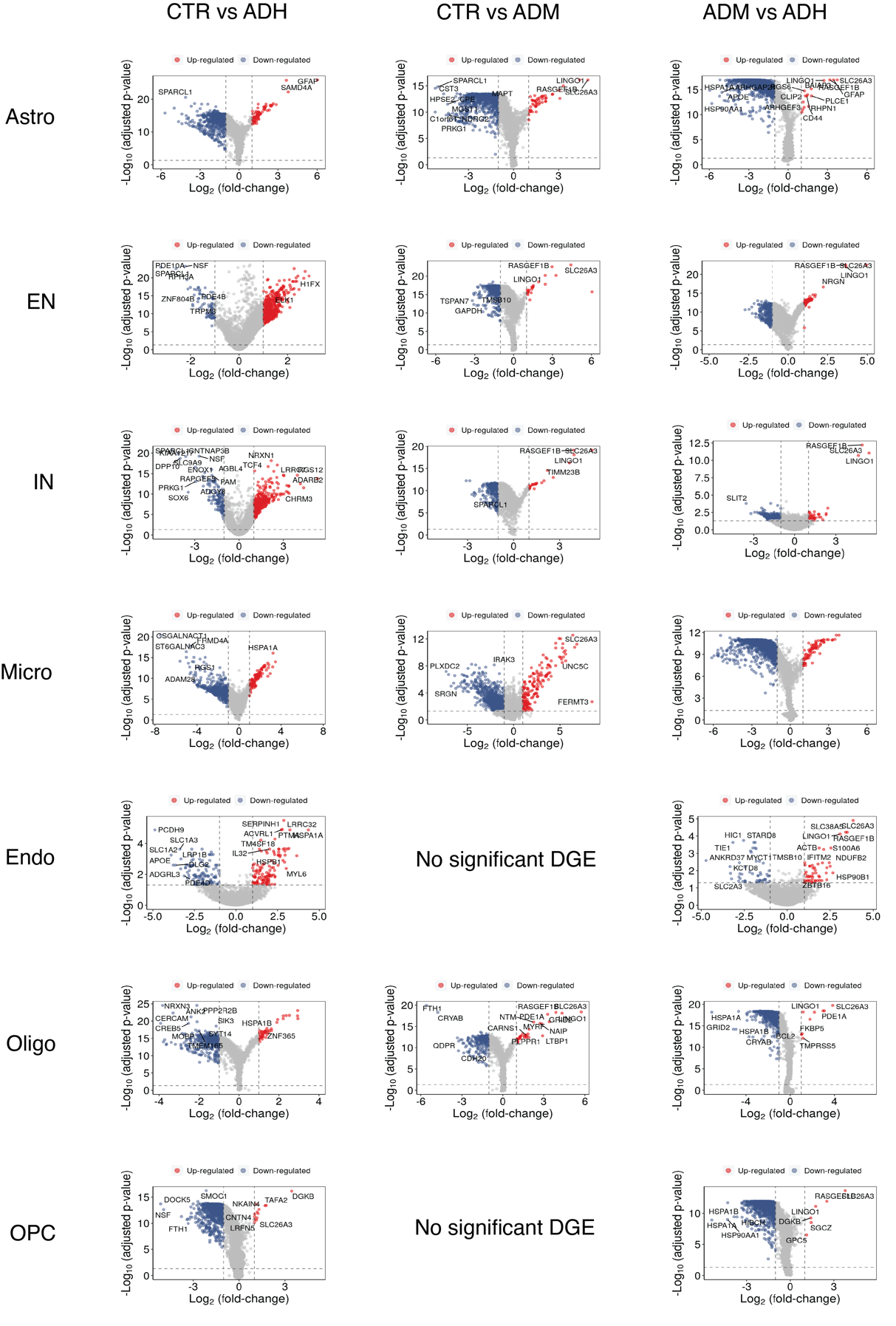


**Supplementary Figure S10** Volcano plots for differential gene expression in NGE (GSE157827) data. Genes with significantly increased expression (red), genes with significantly decreased expression (blue), genes without significant changes (gray), and dashed lines indicate the thresholds of significant changes.


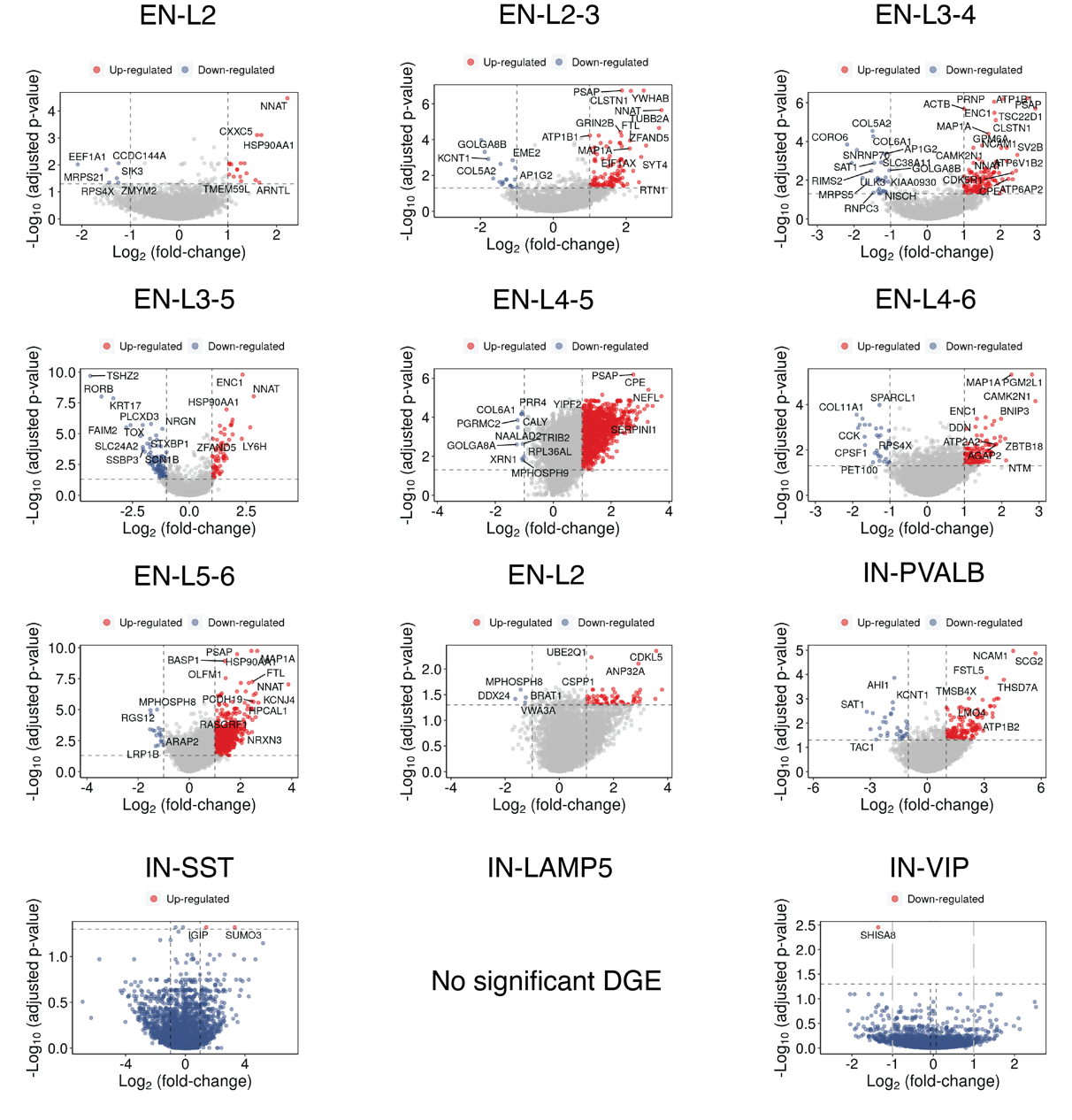


**Supplementary Figure S11** Volcano plots for differential gene expression in NFT (GSE129308) data. Genes with significantly increased expression (red), genes with significantly decreased expression (blue), genes without significant changes (gray) and dashed lines indicate the thresholds of significant changes.

## Supplementary tables

**S1:** doners metadata and covariances

**S2:** ASE of bulk data

**S3:** DTU of bulk data

**S4:** DGE of bulk data

**S5:** ASE and APA in NGE data

**S6:** ASE and APA in NFT data

**S7:** DGE of cell subtypes in NGE and NFT data

The xlsx files of the tables were deposited in supplementary file 2 zip.
